# Self-Setting Calcium Orthophosphate Formulations

**DOI:** 10.3390/jfb4040209

**Published:** 2013-11-12

**Authors:** Sergey V. Dorozhkin

**Affiliations:** Kudrinskaja sq. 1-155, Moscow 123242, Russia; E-Mail: sedorozhkin@yandex.ru; Tel. +7-499-255-4460

**Keywords:** calcium orthophosphates, hydroxyapatite, self-setting, self-hardening, cements, concretes, bioceramics, bone grafts, scaffolds, tissue engineering

## Abstract

In early 1980s, researchers discovered self-setting calcium orthophosphate cements, which are bioactive and biodegradable grafting bioceramics in the form of a powder and a liquid. After mixing, both phases form pastes, which set and harden forming either a non-stoichiometric calcium deficient hydroxyapatite or brushite. Since both of them are remarkably biocompartible, bioresorbable and osteoconductive, self-setting calcium orthophosphate formulations appear to be promising bioceramics for bone grafting. Furthermore, such formulations possess excellent molding capabilities, easy manipulation and nearly perfect adaptation to the complex shapes of bone defects, followed by gradual bioresorption and new bone formation. In addition, reinforced formulations have been introduced, which might be described as calcium orthophosphate concretes. The discovery of self-setting properties opened up a new era in the medical application of calcium orthophosphates and many commercial trademarks have been introduced as a result. Currently such formulations are widely used as synthetic bone grafts, with several advantages, such as pourability and injectability. Moreover, their low-temperature setting reactions and intrinsic porosity allow loading by drugs, biomolecules and even cells for tissue engineering purposes. In this review, an insight into the self-setting calcium orthophosphate formulations, as excellent bioceramics suitable for both dental and bone grafting applications, has been provided.

## 1. Introduction

According to the statistics, approximately half of the population sustains at least one bone fracture during their lifetime [1] and, as a result, surgery might be necessary. Luckily, among the surgical procedures available, minimally invasive techniques are able to offer special benefits for patients such as fewer associated injuries, quicker recovery and less pain. In addition, shorter hospital stays are needed, often allowing outpatient treatments that cheapen the expenses [2]. However, these techniques require biomaterials able to be implanted through small (the smaller, the better) incisions, e.g., by means of syringes with needles and/or laparoscopic devices. To fulfill such requirements, the potential implants should be in a liquid or an injectable state, e.g., as pastes. On the other hand, since all types of the calcified tissues are in the solid state, the bone repairing biomaterials should be solid as well. Therefore, potential bone grafts applicable to the minimally invasive surgery must combine injectability with hardness. Such formulations are known as self-setting (self-hardening, self-curing) formulations because, together with an initial softness and injectability, they possess an ability to solidify in the appropriate period, giving strength to the implantation sites. Since the inorganic part of the mammalian calcified tissues is composed of calcium orthophosphates of biological origin [3], self-setting formulations based on calcium orthophosphates appear to be excellent candidates for bone repairing [4,5]. The list of all known calcium orthophosphates, including their chemical formulae, standard abbreviations and the major properties, is summarized in Table 1 [6].

Although the entire subject of calcium orthophosphates has been investigated since 1770s [7,8], historically, Kingery appears to be the first, who contributed to their self-setting abilities. Namely, in 1950, he published a paper on the chemical interactions between oxides and/or hydroxides of various metals (including CaO) with H_3_PO_4_, in which he mentioned that some of the reaction products were set [9]. However, the calcium orthophosphate formulations were just a very small section of that study. Afterwards, self-setting abilities of some calcium orthophosphates formulations were described in the early 1970s by Driskell *et al*. [10]. However, that study was not noticed. Then, in early 1980s, scientists from the American Dental Association LeGeros *et al*. [11], as well as Brown and Chow [12,13,14,15] published results of their studies. Since that, this subject became known as calcium phosphate cements (commonly referred to as CPC) [16], and, due to their suitability for repair, augmentation and regeneration of bones, such formulations were also named as calcium phosphate bone cements (occasionally referred to as CPBC) [17,18,19,20]. In order to stress the fact, that these formulations consist either entirely or essentially from calcium orthophosphates, this review is limited to consideration of calcium orthophosphate-based compositions only. The readers interested in self-setting formulations based on other types of calcium phosphates are requested to read the original publications [20,21].

**Table 1:** Existing calcium orthophosphates and their major properties [6].

Ca/P molar ratio	Compounds and their typical abbreviations	Chemical formula	Solubility at 25 °C, −log(*K_s_*)	Solubility at 25 °C, g/L	pH stability range in aqueous solutions at 25 °C
0.5	Monocalcium phosphate monohydrate (MCPM)	Ca(H_2_PO_4_)_2_·H_2_O	1.14	~18	0.0–2.0
0.5	Monocalcium phosphate anhydrous (MCPA or MCP)	Ca(H_2_PO_4_)_2_	1.14	~17	^[c]^
1.0	Dicalcium phosphate dihydrate (DCPD), mineral brushite	CaHPO_4_·2H_2_O	6.59	~0.088	2.0–6.0
1.0	Dicalcium phosphate anhydrous (DCPA or DCP), mineral monetite	CaHPO_4_	6.90	~0.048	^[c]^
1.33	Octacalcium phosphate (OCP)	Ca_8_(HPO_4_)_2_(PO_4_)_4_·5H_2_O	96.6	~0.0081	5.5–7.0
1.5	α-Tricalcium phosphate (α-TCP)	α-Ca_3_(PO_4_)_2_	25.5	~0.0025	^[a]^
1.5	β-Tricalcium phosphate (β-TCP)	β-Ca_3_(PO_4_)_2_	28.9	~0.0005	^[a]^
1.2–2.2	Amorphous calcium phosphates (ACP)	Ca *_x_*H*_y_*(PO_4_)*_z_*·*n*H_2_O, *n* = 3–4.5; 15%–20% H_2_O	^[b]^	^[b]^	~5–12 ^[d]^
1.5–1.67	Calcium-deficient hydroxyapatite (CDHA or Ca-def HA) ^[e]^	Ca_10− *x*_(HPO_4_)*_x_*(PO_4_)_6−*x*_(OH)_2−*x*_ (0 < *x* < 1)	~85	~0.0094	6.5–9.5
1.67	Hydroxyapatite (HA, HAp or OHAp)	Ca_10_(PO_4_)_6_(OH)_2_	116.8	~0.0003	9.5–12
1.67	Fluorapatite (FA or FAp)	Ca_10_(PO_4_)_6_F_2_	120.0	~0.0002	7–12
1.67	Oxyapatite (OA, OAp or OXA) ^[f]^	Ca_10_(PO_4_)_6_O	~69	~0.087	^[a]^
2.0	Tetracalcium phosphate (TTCP or TetCP), mineral hilgenstockite	Ca_4_(PO_4_)_2_O	38–44	~0.0007	^[a]^

^[a]^ These compounds cannot be precipitated from aqueous solutions; ^[b]^ Cannot be measured precisely. However, the following values were found: 25.7 ± 0.1 (pH = 7.40), 29.9 ± 0.1 (pH = 6.00), 32.7 ± 0.1 (pH = 5.28). The comparative extent of dissolution in acidic buffer is: ACP >> α-TCP >> β-TCP > CDHA >> HA > FA; ^[c]^ Stable at temperatures above 100 °C; ^[d]^ Always metastable; ^[e]^ Occasionally, it is called “precipitated HA (PHA)” and ^[f]^ Existence of OA remains questionable.

Due to a good bioresorbability, all self-setting calcium orthophosphate formulations belong to the second generation of bone substituting biomaterials [22]. These formulations are blends of amorphous and/or crystalline calcium orthophosphate powder(s) with an aqueous solution, which might be distilled water [11,12,13,14,15], phosphate buffer solution (PBS) [16], aqueous solutions of sodium orthophosphates [23,24,25,26,27,28,29,30], ammonium orthophosphates [31], H_3_PO_4_ [32,33,34,35,36,37], citric acid [24,38] and its salts [39], sodium silicate [40,41,42], soluble magnesium orthophosphates [43], chitosan lactate in lactic acid [44], *etc*. Due to the presence of other ions in a number of solutions, some of such formulations are set with formation of ion-substituted calcium orthophosphates. After the calcium orthophosphate powder(s) and the solution have been mixed together, a viscous and moldable paste is formed that sets to a firm mass within a few minutes. When the paste becomes sufficiently stiff, it can be placed into a defect as a substitute for the damaged part of bone, where it hardens *in situ* within the operating theatre. The proportion of solid to liquid or the powder-to-liquid (P/L) ratio is a very important characteristic because it determines both bioresorbability and rheological properties. As the paste is set and hardened at room or body temperature, direct application in healing of bone defects became a new and innovative treatment modality by the end of the XX-th century. Moreover, self-setting calcium orthophosphate formulations can be injected directly into the fractures and bone defects, where they intimately adapt to the bone cavity regardless its shape. More to the point, they were found to promote development of osteoconductive pathways, possess sufficient compressive strengths, be non-cytotoxic, create chemical bonds to the host bones, restore contour and have both the chemical composition and X-ray diffraction patterns similar to those of bone [45]. Finally, but yet importantly, the self-setting calcium orthophosphate formulations are osteotransductive, *i.e*., after implantation, the hardened formulations are replaced by a new bone tissue [46,47,48].

Since the hardened calcium orthophosphates reproduce the composition, structure, morphology and crystallinity of bone crystals, the initial self-setting formulations might be considered as biomimetic ones [49,50]. The aim of such formulations is to disturb bone functions and properties as little as possible and, until a new bone has been grown, to behave temporary in a manner similar to that of bone. Therefore, they provide surgeons with a unique ability of manufacturing, shaping and implanting the bioactive bone substitute biomaterials on a patient-specific base in real time in the surgery room. Implanted bone tissues also take benefits from the self-setting formulations that give, in an acceptable clinical time, a suitable mechanical strength for a shorter tissue functional recovery. Thus, the major advantages of the self-setting calcium orthophosphate formulations include a fast setting time, an excellent moldability, an outstanding biocompatibility and an easy manipulation; therefore, they are more versatile in handling characteristics than prefabricated granules or blocks. Besides, like any other type of calcium orthophosphate bioceramics, the self-setting formulations provide an opportunity for bone grafting using alloplastic materials, which are unlimited in quantity and provide no risk of infectious diseases [51,52,53].

Since self-setting calcium orthophosphate formulations have been developed for using as implanted biomaterials for parenteral application, for their chemical composition one might employ all ionic compounds of oligoelements occurring naturally in a human body. The list of possible additives includes (but is not limited to) the following cations: Na^+^, K^+^, Mg^2+^, Ca^2+^, Sr^2+^, Zn^2+^, H^+^ and anions: PO_4_^3−^, HPO_4_^2−^, H_2_PO_4_^−^, P_2_O_7_^4−^, CO_3_^2−^, HCO_3_^−^, SO_4_^2−^, HSO_4_^−^, Cl^−^, OH^−^, F^−^, silicates [46]. Therefore, mixed-type self-setting formulations consisting of calcium orthophosphates and other calcium salts, such as calcium sulfate [54,55,56,57,58,59,60,61,62,63], calcium pyrophosphate [64,65,66], calcium polyphosphates [67,68], calcium carbonates [16,26,28,30,50,69,70,71], calcium oxide [72,73,74,75,76,77], calcium hydroxide [78,79,80], calcium aluminates [43,81,82], calcium silicates [83,84,85,86,87,88,89], *etc*., are available. In addition, other chemicals such as Sr-containing compounds [19,90,91,92,93], Mg-containing compounds [93,94,95,96,97,98,99,100], Zn-containing compounds [101,102], *etc*., might be added to calcium orthophosphates as well. Furthermore, the self-setting formulations might be prepared from various types of ion substituted calcium orthophosphates such as Ca_2_KNa(PO_4_)_2_, NaCaPO_4_, Na_3_Ca_6_(PO_4_)_5_ (so called “calcium alkaline orthophosphates”) [103,104,105,106,107], magnesium substituted calcium-deficient hydroxyapatite (CDHA), strontium substituted CDHA, *etc*. [108,109,110,111,112,113]. More to the point, self-setting formulations might be prepared in the reaction-setting mixture of Ca(OH)_2_–KH_2_PO_4_ system [114], as well as by treatment of calcium carbonate or calcium hydroxide with orthophosphate solutions [115]. In addition, if a self-setting formulation consisting of calcium orthophosphates only is set in a chemically reactive environment (e.g., in presence of CO_2_), ion-substituted calcium orthophosphates, such as carbonate apatite, are formed [116]. Finally, self-setting calcium orthophosphate-based formulations possessing special properties, such as magnetic ones due to incorporation of iron oxides [117,118] have been developed as well. However, with a few important exceptions, the ion-substituted formulations have not been considered in this review, while the interested readers are suggested to study the aforementioned publications.

The purpose of this review is to evaluate the chemistry, physical, mechanical and biomedical properties of the available self-setting calcium orthophosphate formulations with the specific reference to their applications in surgery and dentistry.

## 2. General Information and Knowledge

According to Wikipedia, the free encyclopedia: “In the most general sense of the word, cement is a binder, a substance that sets and hardens independently and can bind other materials together”. The name “cement” goes back to the Romans who used the term “*opus caementitium*” to describe masonry, which resembled concrete and was made from crushed rock with burnt lime as binder. The volcanic ash and pulverized brick additives, which were added to the burnt lime to obtain a hydraulic binder, were later referred to as cementum, cimentum, cäment and cement” [119]. Thus, calcium orthophosphate cement appears to be a generic term to describe chemical formulations in the ternary system Ca(OH)_2_–H_3_PO_4_–H_2_O which can experience a transformation from a liquid or a pasty state to a solid state and in which the end-product of the chemical reactions is a calcium orthophosphate.

**Figure 1:**
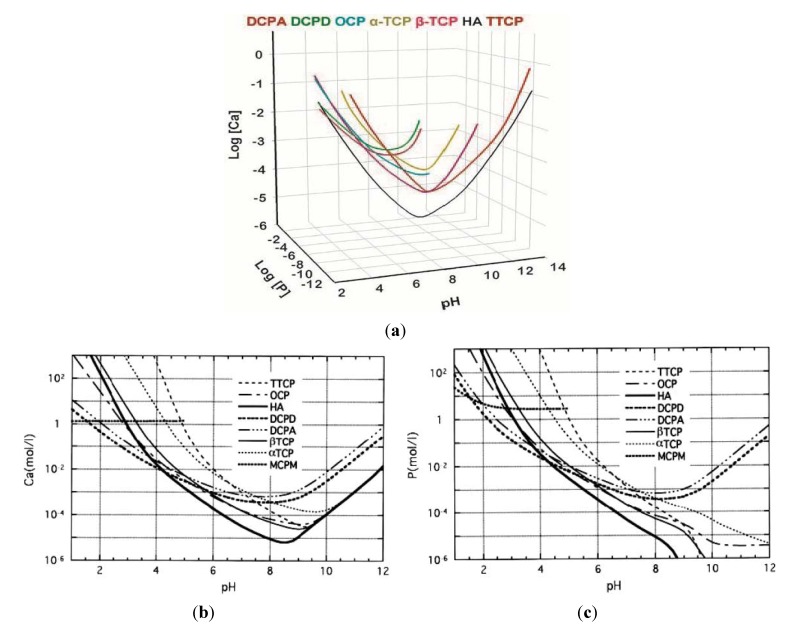
(**a**): a 3D version of the classical solubility phase diagrams for the ternary system Ca(OH)_2_–H_3_PO_4_–H_2_O. Reprinted from [150] with permission. (**b**,**c**): solubility phase diagrams in two-dimensional graphs, showing two logarithms of the concentrations of (a) calcium and (b) orthophosphate ions as a function of the pH in solutions saturated with various salts. Reprinted from [151] with permission.

The first self-setting calcium orthophosphate formulation consisted of the equimolar mixture of TTCP and dicalcium phosphate (DCPA or DCPD) which was mixed with water at a P/L ratio of 4:1; the paste hardened in about 30 min and formed CDHA. These highly viscous and non-injectable pastes could be molded and, therefore, were used mainly as a contouring material in craniofacial surgery. Later studies revealed some differences between TTCP + DCPD and TTCP + DCPA formulations. Namely, due to a higher solubility of DCPD (Table 1 and Figure 1), TTCP + DCPD mixtures set faster than TTCP + DCPA ones. Besides, injectability of TTCP + DCPD formulations is better [120,121,122]. In 1990s, it was established that there were about 15 different binary combinations of calcium orthophosphates, which gave self-setting pastes upon mixing with water or aqueous solutions. The list of these combinations is available in literature [123,124,125]. From these basic systems, secondary self-setting formulations could be derived containing additional or even non-reactive compounds [17,46,74,123,126,127,128,129,130,131,132,133,134,135,136,137,138,139]. Concerning their viscosity, both pasties [140,141,142,143,144,145] and putties [146] of a very high viscosity [146,147,148,149] are known.

According to the classical solubility data of calcium orthophosphates (Figure 1), depending upon the pH value of a self-setting paste, after hardening all formulations can form only two major end-products: a precipitated poorly crystalline HA or CDHA at pH > 4.2 and DCPD (also called “brushite”) at pH < 4.2 [152]. Here one should notice, that in the vast majority cases, terms “a precipitated poorly crystalline HA” and “CDHA” are undistinguishable and might be considered as synonyms [6], while the term “brushite” was coined to honor an American mineralogist George Jarvis Brush (1831–1912), who was a professor at Yale University, USA. However, in the real self-setting formulations, the pH-border of 4.2 might be shifted to higher pH values. Namely, DCPD might be crystallized at the solution pH up to ~6, while CDHA normally is not formed at pH below 6.5–7 (Table 1).

In early 1990s, depending on the type of calcium orthophosphate formed after the setting, five groups of the self-setting formulations were thought to exist: DCPD, CDHA, HA, ACP and OCP [125,153], while currently only two cement groups remain. Namely, the results of the only study on an ACP-forming formulation demonstrated that it was rapidly converted into CDHA [137]; thus, it belongs to apatite-forming formulations. With the OCP-forming formulations [154,155,156,157] the situation looks as this. Contrary to the reports of late 1980s [154] and early 1990s [155], in recent papers either simultaneous formation of OCP and CHDA has been detected [157] or no phase analysis has been performed [156]. Strong experimental evidences of the existence of a transient OCP phase during setting were found in still another study; however, after a few hours, the OCP phase disappeared giving rise to the final CDHA phase [41]. Finally, according to the aforementioned, CDHA and HA are synonyms. Thus, all existing self-setting calcium orthophosphate formulations are divided into two major groups: apatite-forming formulations and brushite-forming ones. This in fact is a predictable situation since HA is the least soluble calcium orthophosphate at pH > 4.2 and brushite is the least soluble one at pH < 4.2 (Figure 1). The final hardened product of the formulations is of the paramount importance because it determines the solubility and, therefore, *in vivo* bioresorbability. Since the chemical composition of mammalian bones is similar to an ion-substituted CDHA, apatite-forming formulations have been more extensively investigated. Nevertheless, many research papers on brushite-forming formulations have been published as well.

**Figure 2:**
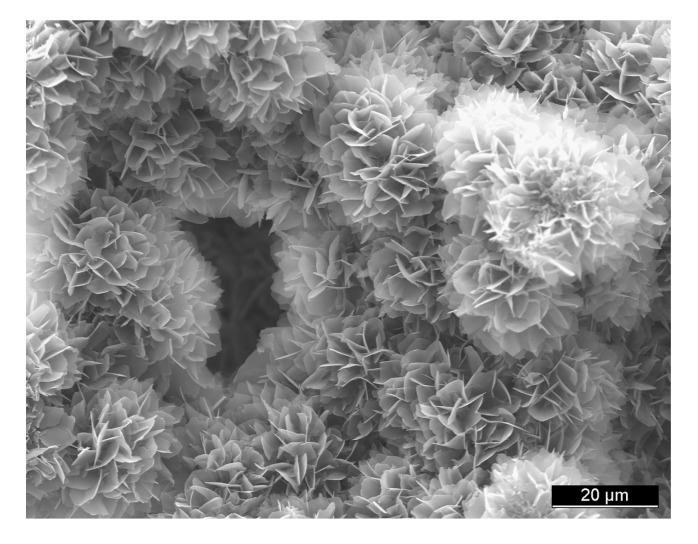
A typical microstructure of calcium orthophosphate formulation after hardening. The mechanical stability is provided by the physical entanglement of crystals. Reprinted from [161] with permission.

All self-setting calcium orthophosphate formulations are made of an aqueous solution and fine powders of one or several calcium orthophosphate(s). Here, dissolution of the initial calcium orthophosphate(s) (quickly or slowly depending on the chemical composition and solution pH) and mass transport appear to be the primary functions of an aqueous environment, in which the dissolved reactants form a supersaturated (very far away from the equilibrium) microenvironment with regard to precipitation of the final product(s) [158,159]. The relative stability and solubility of various calcium orthophosphates (see Table 1) is the major driving force of the setting reactions occurred. Therefore, mixing of a dry powder with an aqueous solution induces various chemical transformations, in which crystals of the initial calcium orthophosphate(s) rapidly dissolve(s) and precipitate(s) into crystals of CDHA (precipitated HA) or DCPD with possible formation of intermediate precursor phases (e.g., ACP [30,137] and OCP [41,154,155,156,157]). During precipitation, the newly formed crystals grow and form a web of intermingling microneedles or microplatelets of CDHA or DCPD, thus provide a mechanical rigidity to the hardened cements. In other words, entanglement of the newly formed crystals is the major reason of setting (Figure 2). For the majority of apatite-forming formulations, water is not a reactant in the setting reactions; it is just a medium for reactions to occur. Therefore, the quantity of water, actually needed for setting of such formulations, is very small [22,158,160]. However, for the brushite-forming formulations, water always participates in the chemical transformations because it is necessary for DCPD formation. Due to this reason, the brushite-forming formulations are always hydraulic, while usually this term is not associated with the apatite-forming ones.

Setting of calcium orthophosphate formulations is a continuous process that always starts with dissolution of the initial compounds in an aqueous system. This supplies ions of calcium and orthophosphate into the solution, where they chemically interact and precipitate in the form of either the final products or precursor phases, which causes the cement setting [13,162,163]. This was confirmed by Ishikawa and Asaoka, who showed that when TTCP and DCPA powders were mixed in double-distilled water, both powders were dissolved. The dissolved calcium and orthophosphate ions in the solution were then precipitated in the form of CDHA on the surface of unreacted powders [164]. Since the physical state of the precipitates can be either a gel or a conglomerate of crystals, the hardening mechanism is either a sol-gel transition of ACP [30,137] or entanglement of the precipitated crystals of CDHA or DCPD [46]. Thus, all types of hardened formulations possess an intrinsic porosity within the nano/submicron size ranges (Figure 2). For example, for the classical Brown-Chow cement formulation, after the initial setting, petal or needle-like crystals enlarge epitaxially and are responsible for the adherence and interlocking of the crystalline grains, which result in hardening. After ~2 h, the newly formed crystals become rod-like, resulting from higher crystallinity with the observation of more material at the inter-particle spaces. During this period, the setting reactions proceeded at a near-constant rate, suggesting that the reaction rate was limited by factors that are unrelated to the amounts of the starting materials and the reaction products present in the system. Such factors could be related to the surface area of DCPA or TTCP or to the diffusion distances over which the calcium and orthophosphate ions should migrate to form CDHA [165,166,167]. At ~24 h, the crystals are completely formed, being very compacted in some areas of high density, and well separated in areas with more porosity [130,135,136].

The chemical reactions occurring during setting of calcium orthophosphate formulations depend on their chemical composition. However, it can be stated that only two major chemical types of the setting reaction are possible. The first type occurs according to the classical rules of the acid-base interaction, *i.e*., a relatively acidic calcium orthophosphate reacts with a relatively basic one to produce a relatively neutral compound. The first cement by Brown and Chow is a typical example of this type because TTCP (basic) reacts with DCPA (slightly acidic) in an aqueous suspension to form a poorly crystalline precipitated HA (slightly basic) [13,14]:

2Ca_4_(PO_4_)_2_O + 2CaHPO_4_ = Ca_10_(PO_4_)_6_(OH)_2_ (1)

Initially, it was believed that DCPA and TTCP reacted upon mixing with water to form the stoichiometric HA [12,13,14,15]. However, further investigations have shown that only the first nuclei consist of a nearly stoichiometric HA, whereas further growth of these nuclei occurs in the form of CDHA [168]. Besides, there is a study demonstrating that the initially formed stoichiometric HA further interacts with remaining DCPD to form CDHA [169].

According to Equation (1), formation of precipitated HA releases neither acidic nor basic by-products. Thus, the liquid phase of the formulation remains at a near constant pH of ~7.5 for the TTCP + DCPD and ~8.0 for the TTCP + DCPA mixtures, respectively [165,166,167]. Various deviations from the stoichiometry of chemical Equation (1) were studied in details and various types of CDHA with Ca/P ionic ratio within 1.5–1.67 were found as the final product [170]. The effect of mixing ratio and pH on the reaction between TTCP and DCPA is well described elsewhere [171]. Furthermore, the influence of Ca/P ionic ratio of TTCP on the properties of the TTCP + DCPD cement was studied as well [172].

A blend proposed by Lema < ître *et al*., [173,174] is another example of the acid-base interaction in which β-TCP (almost neutral) reacts with MCPM (acidic) to form DCPD (slightly acidic):

β-Ca_3_(PO_4_)_2_ + Ca(H_2_PO_4_)_2_·H_2_O + 7H_2_O = 4CaHPO_4_·2H_2_O (2)

In chemical Equation (2) MCPM might easily be substituted by H_3_PO_4_ [32,33,34,35,36] or MCPA, while β-TCP might be replaced by α-TCP [175,176], CDHA [177,178], HA [179,180] or even Ca(OH)_2_ [29,35] and CaO. For example:

Ca_9_(HPO_4_)(PO_4_)_5_(OH) + 3H_3_PO_4_ + 17H_2_O = 9CaHPO_4_·2H_2_O (3)

Furthermore, self-setting formulations based on mixtures of ACP + α-TCP [181], ACP + DCPD [182,183], DCPA + α-TCP [176], OCP + TTCP [184], OCP + α-TCP [185,186] and unspecified “partially crystallized calcium phosphate” (presumably, CDHA) + DCPA [187,188,189] as the initial reagents, are also available. In addition, multiphase self-setting compositions such as α-TCP + TTCP + DCPA [190] and DCPA + α-TCP + β-TCP + CDHA [191] have been developed as well.

The second type of the setting reaction might be defined as hydrolysis of metastable calcium orthophosphates in aqueous media. As the result, both the initial components and final products have the same Ca/P ionic ratio. Due to the fact, that only one calcium orthophosphate is used; the solid part of such formulations might be called as a single-phase (or single-component) cement powder [192]. Self-setting formulations made of ACP + an aqueous solution [193,194], α-TCP + an aqueous solution [23,24,27,195,196,197,198,199,200,201,202,203], β-TCP + an aqueous solution [199,204], DCPA + an aqueous solution [40], CDHA + an aqueous solution [41], OCP + an aqueous solution [42], TTCP + an aqueous solution [43,205,206] or γ-radiated TTCP + an aqueous solution [207,208,209] are the typical examples; the majority of them are re-crystallized to CDHA during setting:

Ca*_x_*H*_y_*(PO_4_)*_z_*·*n* H_2_O + H_2_O → Ca_10-*x*_(HPO_4_)*_x_*(PO_4_)_6-*x*_(OH)_2-*x*_ + *n* H_2_O (4)

3(α- or β-)Ca_3_(PO_4_)_2_ + H_2_O = Ca_9_(HPO_4_)(PO_4_)_5_(OH) (5)

3Ca_4_(PO_4_)_2_O + 3H_2_O = Ca_9_(HPO_4_)(PO_4_)_5_(OH) + 3Ca(OH)_2_ (6)

As seen from the amount of publications, α-TCP is the most popular compound to produce self-setting single-phase calcium orthophosphate formulations.

An interesting study was performed on the microstructures, mechanical and setting properties of calcium orthophosphate formulations with variable Ca/P ratio within 1.29 < Ca/P < 1.77 [210]. The results showed that: (a) only the reactant with Ca/P = 1.50 was monophasic and consisted of α-TCP, which transformed during the setting into CDHA; (b) reactants with Ca/P < 1.50 were composed of calcium pyrophosphate, α-TCP and β-TCP blends, while those with Ca/P > 1.50 were composed of α-TCP, HA and TTCP blends; (c) formulations with Ca/P ratio other than 1.50 had longer setting and lower hardening properties; (d) the formulations’ reactivity was clearly affected by the Ca/P ratio of the starting reactant; (e) depending on the Ca/P ratio of the starting reactant, the hardened formulations developed different crystal microstructures with specific features [210]. Similarly, a self-setting formulation might be prepared from the thermal decomposition products of HA [211].

**Figure 3:**
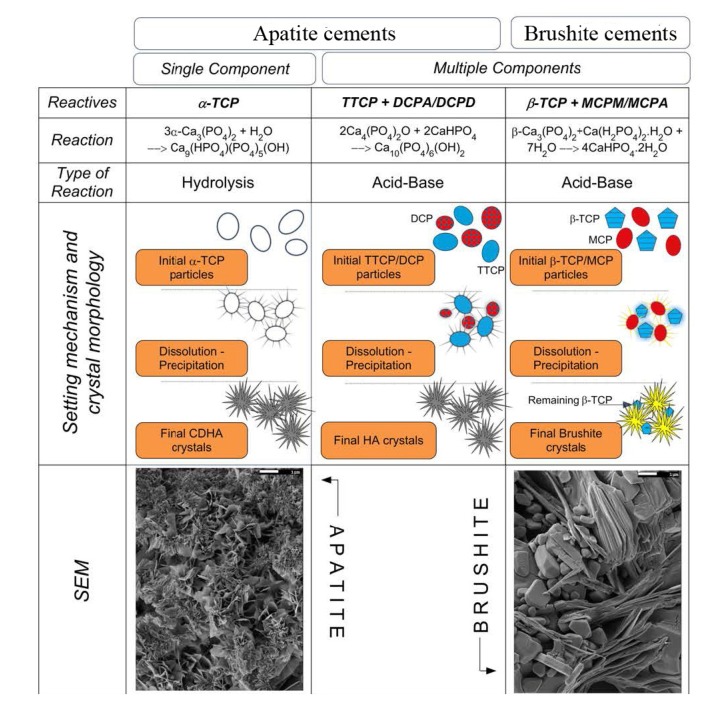
Add Classification of calcium orthophosphate formulations with examples of the most common compositions. Scanning electron micrographs of set apatite and brushite cements obtained by the hydrolysis of α-Tricalcium phosphate (α-TCP) and by reaction of β-Tricalcium phosphate (β-TCP) with monocalcium phosphate monohydrate (MCPM), respectively, are also shown. Reprinted from [219] with permission.

The experimental details on TTCP hydrolysis under a near-constant composition condition might be found elsewhere [212]. The details on α-TCP hydrolysis are also available. The results indicated that setting of α-TCP was initially controlled by surface dissolution; therefore, it depended on the surface area of the reactants [213,214,215,216]. Hydrolysis of DCPD to CDHA was studied as well [217]. Addition of ~2 wt% of a precipitated poorly crystalline HA (*i.e*., CDHA) as a seed to α-TCP powder phase might be useful to accelerate the kinetics of reaction (5) [218]. The aforementioned information is summarized in Figure 3 [219].

Further, there is a single-phase formulation consisting of K- and Na- containing CDHA (with the Ca/P ionic ratio of 1.64 ± 0.02) that sets and hardens after mixing with an aqueous solution of sodium citrate and sodium orthophosphate [220]. After setting, this formulation gives rise to formation of a weak cement (the compressive strength of 15 ± 3 MPa) consisting of the ion-substituted CDHA again (presumably, with smaller Ca/P ionic ratio), mimicking the bone mineral. Unfortunately, neither the setting reaction nor the setting mechanism of this cement has been disclosed [220].

The hydration process of calcium orthophosphate formulations is slightly exothermic and undergoes five periods: initiating period, induction period, accelerating period, decelerating period and terminating period [221]. For the classical Brown-Chow formulation, the activation energy of the hydration reaction is 176 kJ/mol [222]. The rate of heat liberation during the solidification of calcium orthophosphate formulations is low. The results of adiabatic experiments showed that the temperature rise arrived at the highest value of 37 °C 3 h later, which would cause no harm to surrounding tissues [221]. The results showed that the hardening process of that formulation was initially controlled by dissolution of the reactants in a 4 h period and subsequently by diffusion through the product layer of CDHA around the grains [136]. In general, setting of calcium orthophosphate formulations occurs mostly within the initial ~6 h, yielding an ~80% conversion to the final products with the volume almost constant during setting (*i.e*., shrinkage is small). However, after hardening, the formulations always form brittle bioceramics with the tensile strength of 5 to 20 times lower than the compression strength [223,224]. Since this biomaterial is weak under tensile forces, such formulations can only be used either in combination with metal implants or in non-load bearing (e.g., craniofacial) applications [4,5,160,225]. This is confirmed by the mechanical characterization of a bone defect model filled with bioceramic cements [226].

To conclude this part, one must stress, that chemical Equations (1)–(6) for setting processes are valid for the *in vitro* conditions only. There are evidences that samples of calcium orthophosphate formulations retrieved 12 h after hardening *in vivo* already contained carbonate apatite, even though the initial mixtures did not contain carbonate as one of the solid components [227]. The mass fraction of carbonate in the 12 h samples was about 1%. The results suggest that under the *in vivo* conditions, carbonate is readily available and this allows formation of carbonate apatite in favor of carbonate-free CDHA [227].

**Figure 4:**
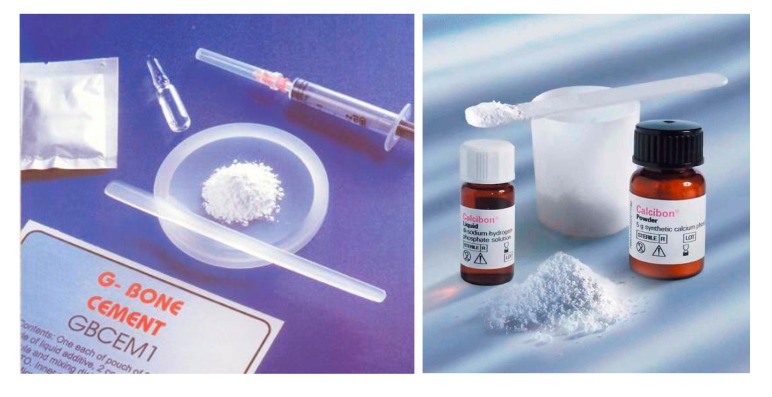
A presentation of two randomly chosen commercial calcium orthophosphate cements.

By the end of the previous millennium, the United States Food and Drug Administration (FDA) approved several self-setting formulations (Table 2) for clinical use [22,228]. The same formulations also received a Conformite Europene (CE) mark for certain maxillofacial indications and for use as bone-void fillers in the specific non-load-bearing orthopedic indications [160]. The major properties of these formulations are available in literature [22]. An extended list of the available formulations is presented in Table 3 [149], while even more formulations are in experimental stages. Other lists of the commercially available injectable bone cements with their chemical composition (when obtainable) might be found elsewhere [5,167,229,230,231]. A general appearance of two randomly chosen commercial calcium orthophosphate cements is shown in Figure 4.

**Table 2:** Some self-setting calcium orthophosphate formulations having the 510(k) clearance from the Food and Drug Administration (FDA) [17,160,228]. The technical data on these cements might be found in literature [22].

Product *	Manufacturer	Applications *
BoneSource^TM^ **	Striker Howmedica Osteonics (Rutherford, NJ, USA)	Craniofacial
α-Bone Substitute Material (α-BSM^®^) ***	Etex Corporation (Cambridge, MA, USA)	Filling of bone defects and voids, dental, craniofacial
Skeletal Repair Systems (SRS^®^)	Norian Corporation (Cupertino, CA, USA)	Skeletal distal radius fractures, craniofacial

* In Europe, other applications may apply, and the materials may be sold with a different commercial name; ** BoneSource^TM^ is the original formulation of calcium orthophosphate cement developed by Brown and Chow; *** In Europe, it is distributed by Biomet Merck (Zwijndrecht, The Netherlands) as Biobon^®^ [160], while in North America it is marketed by Walter Lorenz Surgical (Jacksonville, FL, USA) as Embarc^®^ [22].

**Table 3:** A list of the commercial self-setting calcium orthophosphate formulations with the producer, product name, composition (when available) and main end-product. The end-product of the reactions can be either an apatite (CDHA, carbonate apatite, *etc*.) or brushite (=DCPD) [149].

Producer	Commercial name	Composition			Product
aap Implantate (GER)	OsteoCem^®^	Powder: calcium orthophosphates (details unknown); Solution: unknown			apatite
Berkeley Advanced Biomaterials (U.S.)	Cem-Ostetic^TM^	Powder: calcium orthophosphates (details unknown); Solution: water			apatite
	Tri-Ostetic^TM^	Powder: calcium orthophosphates (details unknown); Solution: water			apatite
Biomatlante (FR)	MCPC	Powder: mainly α-TCP, ACP, BCP (HA + β-TCP); Solution: phosphate buffered solution			apatite
Biomet (U.S.) Interpore (U.S.)	Calcibon^®^	Powder: α-TCP (61%), DCPA (26%), CaCO_3_ (10%), CDHA (3%); Solution: H_2_O, Na_2_HPO_4_			apatite
Walter Lorenz Surgical (GER)	Mimix^TM^	Powder: TTCP, α-TCP, trisodium citrate; Solution: citric acid aqueous solution			apatite
	Quick Set Mimix^TM^	Powder: Calcium orthophosphate powders, trisodium citrate; Solution: citric acid aqueous solution			apatite
Calcitec (U.S.)	Osteofix	Powder: calcium orthophosphate and calcium oxide powders; Solution: phosphate buffer			apatite
ETEX (U.S.)	α-BSM^®^; Embarc; Biobon	Powder: ACP (50%), DCPD (50%); Solution: un-buffered aqueous saline solution			apatite
	β-BSM^®^	Composition: could not be found (it has apparently a higher compressive strength and better injectability than α-BSM^®^)			apatite
	γ-BSM^®^	Composition: could not be found (putty consistency)			apatite
	OssiPro	Composition: could not be found; the cement is claimed to be macroporous after hardening			apatite
	CarriGen	Composition: synthetic calcium orthophosphate, sodium carboxymethylcellulose, sodium bicarbonate and sodium carbonate			apatite
Graftys (FR)	Graftys^®^ HBS	Powder: α-TCP (78%), DCPD (5%), MCPM (5%), CDHA (10%), hydroxypropylmethylcellulose (2%); Solution: 5% Na_2_HPO_4_ aqueous solution			apatite
	Graftys^®^ Quickset	Composition: calcium orthophosphate salts, hydroxypropylmethylcellulose and orthophosphate-based aqueous solution			apatite
Kasios (FR)	Jectos Eurobone^®^	Powder: β-TCP (98%), Na_2_P_2_O_7_ (2%); Solution: H_2_O, H_3_PO_4_ (3.0 M), H_2_SO_4_ (0.1 M)			brushite
	Jectos+	Composition: could not be found (likely to be close to that of Jectos)			brushite
Kyphon (U.S.)	KyphOs^TM^		Powder: β-TCP (77%), Mg_3_(PO_4_)_2_ (14%), MgHPO_4_ (4.8%), SrCO_3_ (3.6%); Solution: H_2_O, (NH_4_)_2_HPO_4_ (3.5 M)	apatite	
Merck (GER) Biomet (U.S.)	Biocement D		Powder: 58% α-TCP, 24% DCPA, 8.5% CaCO_3_, 8.5% CDHA; Solution: 4 wt% Na_2_HPO_4_ in water	apatite	
Mitsubishi Materials (J)	Biopex^®^		Powder: α-TCP (75%), TTCP (20%–18%), DCPD (5%), HA (0%–2%) Solution: H_2_O, Na succinate (12%–13%), Na chondroitin sulfate (5%–5.4%)	apatite	
	Biopex^®^-R		Powder: α-TCP, TTCP, DCPD, HA, Mg_3_(PO_4_)_2_, NaHSO_3_; Solution: H_2_O, Na succinate, Na chondroitin sulfate	Apatite	
Produits Dentaires SA (CH) CalciphOs (CH)	VitalOs^4^		Solution 1: β-TCP (1.34 g), Na_2_H_2_P_2_O_7_ (0.025 g), H_2_O, salts (0.05 M PBS solution, pH 7.4); Solution 2: MCPM (0.78 g), CaSO_4_·2H_2_O (0.39 g), H_2_O, H_3_PO_4_ (0.05 M)	Brushite	
Shanghai Rebone Biomaterials Co (CN)	Rebone		Powder: TTCP, DCPA; Solution: H_2_O	Apatite	
Skeletal Kinetics (U.S.)	Callos^TM^		Composition: α-TCP, CaCO_3_, MCPM; Solution: sodium silicate	Apatite	
	Callos Inject^TM^		Composition: α-TCP and unknown compounds (likely to be close to that of Callos^TM^)	Apatite	
	OsteoVation EX Inject		Probably similar to Callos Inject^TM^ (Product produced by S.K. but sold by OsteoMed)	Apatite	
Stryker (U.S.) Leibinger (GER)	BoneSource^TM^		Powder: TTCP (73%), DCPD (27%); Solution: H_2_O, mixture of Na_2_HPO_4_ and NaH_2_PO_4_	Apatite	
Stryker (U.S.)	HydroSet^TM^		Powder: TTCP, DCPD, trisodium citrate; Solution: H_2_O, polyvynilpyrrolidone, Na orthophosphate	Apatite	
DePuy Synthes (U.S.)	Norian^®^ SRS Norian^®^ CRS		Powder: α-TCP (85%), CaCO_3_ (12%), MCPM (3%); Solution: H_2_O, Na_2_HPO_4_	Apatite	
	Norian^®^ SRS Fast Set Putty Norian^®^ CRS Fast Set Putty		Composition: could not be found (likely to be close to that of Norian SRS/CRS)	Apatite	
	Norian Drillable		Composition: calcium orthophosphate powder, bioresorbable fibers and Na hyaluronate solution	Apatite	
	ChronOS^TM^ Inject		Powder: β-TCP (73%), MCPM (21%), MgHPO_4_·3H_2_O (5%), MgSO_4_ (< 1%), Na_2_H_2_P_2_O_7_ (< 1%); Solution: H_2_O, Na hyaluronate (0.5%)	Brushite	
Teknimed (FR)	Cementek^®^		Powder: α-TCP, TTCP, Na glycerophosphate; Solution: H_2_O, Ca(OH)_2_, H_3_PO_4_	Apatite	
	Cementek^®^ LV		Powder: α-TCP, TTCP, Na glycerophosphate, dimethylsiloxane; Solution: H_2_O, Ca(OH)_2_, H_3_PO_4_	Apatite	

## 3. Two Major Types of the Self-Setting Calcium Orthophosphate Formulations

### 3.1. Apatite-Forming Formulations

As indicated by its name, apatite-forming formulations have a poorly crystalline precipitated HA and/or CDHA as the final product of setting reactions [chemical Equations (1) and (4)–(6)], although traces of un-reacted starting compounds can be present [130]. Self-setting FA-forming formulations are also known; they can be prepared by the same way but in the presence of soluble F^−^-ions [37,232,233]. Due to the initial presence of carbonates, such commercial formulations as Norian SRS^®^ and Biocement D^®^ (Table 3) form a non-stoichiometric carbonate apatite or dahllite [Ca_8.8_(HPO_4_)_0.7_(PO_4_)_4.5_(CO_3_)_0.7_(OH)_1.3_] as the end-product [69,234]. As both CDHA and carbonate apatite are formed in an aqueous environment and have a low crystallinity, they appear to be rather similar to the biological apatite of bones and teeth. These properties are believed to be responsible for their excellent *in vivo* resorption characteristics. Conventional apatite-forming formulations contain TCP and/or TTCP phases in their powder components [230], while a single component formulation consisting of K- and Na- containing CDHA is also available [220]. The reactivity of TCP-based apatite-forming formulations was found to vary as a function of TCP crystal phase, crystallinity and particle size [235,236]. Generally, a higher reactivity is observed with a thermodynamically less stable phase (from β-TCP to α-TCP and further to ACP) and with a smaller particle size [199]. Nominally, it might be stated that formation of apatites through self-setting reactions is a sort of a biomimetic process because it occurs in physiological environment and at body temperature [53]; however, both the crystallization kinetics and a driving force are very far away from the biomimeticity. A unique feature of the hardened apatite-forming formulations is that the force linking the newly formed crystals (of both CDHA and carbonate apatite) is weak; therefore, the crystals can be easily detached from the bulk of hardened formulations, especially after dissolution has partly occurred. When this happens, osteoclasts and other cells can easily ingest the apatite crystals [237].

**Figure 5:**
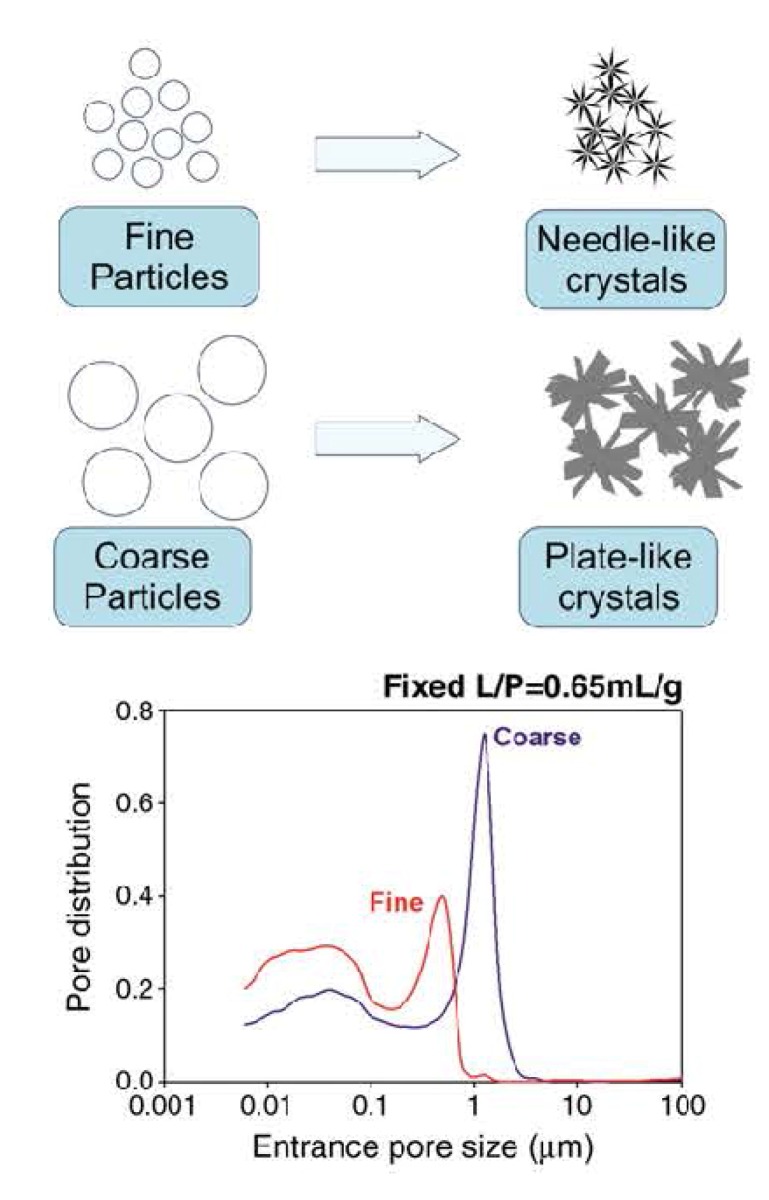
A schematic drawing of the influence of the particle dimensions on the properties of self-setting formulations. Reprinted from [219] with permission.

Immediately after implantation, any formulation becomes exposed to blood and other tissue fluids that delay the setting time. Intrinsic setting time for apatite-forming formulations has been extensively studied and it appears to be rather long. For example, for the original formulation by Brown and Chow it ranges from 15 to 22 min [13,14]. This may result in procedural complications. To remedy this, the amount of liquid might be reduced to a possible minimum. In such cases, all apatite-forming formulations look like viscous and easily moldable pastes, which tend to be difficult to inject. Besides playing with the P/L ratio, the setting time can also be reduced by using additives to the liquid phase (which is distilled water in the Brown-Chow formulation [13,14]). The list of possible additives includes H_3_PO_4_, MCPM and other soluble orthophosphates. These additives promote dissolution of the initial solids by lowering the solution pH. In such cases, a setting time in the range of 10–15 min can be obtained [193,194,195,196,197,198,199,200,201,238]. The influence of soluble orthophosphates (e.g., Na_2_HPO_4_ or NaH_2_PO_4_) on the setting time is explained by the fact that dissolution of DCPA and formation of CDHA during setting occur in a linear fashion, thus avoiding early formation of CDHA. This is important because too early formation of CDHA might engulf un-reacted DCPA, which slows down DCPA dissolution and thus the setting kinetics becomes slower, while the presence of sodium orthophosphates prevents DCPA particles from being isolated [239]. Particle size [218,240,241], temperature and initial presence of HA powders as seeds in the solid phase are other factors that influence the setting time [13,14,53,235,236]; however, *in vitro* studies demonstrated that these parameters did not affect significantly [130]. On the other hand, particle size reduction was found to result in a significant decrease in both initial and final setting times [218,240,241], an acceleration of the hardening rate [218] and hydration kinetics of the hardening formulation [241]. In general, smaller crystals or particles result in a higher supersaturation degrees achieved in the self-setting pastes, which favors crystal nucleation and results in the precipitation of greater many and smaller needle-like crystals, instead of the larger plate-like crystals formed when bigger particles are used (Figure 5) [219]. These different microstructures give rise to different pore size distributions in the set formulations (bottom part of Figure 5). Besides, the crystallite sizes of the final products can be strongly reduced by increasing the specific surface of the starting powders, which allows developing formulations with tailored structures at the micro and nano-scale levels [218]. Unfortunately, an unclear correlation was found between the particle dimensions of the initial calcium orthophosphates and mechanical properties of the hardened products: namely, a significant increase in compressive strength and storage modulus was reported for some formulations [240,241] but a minor effect on compressive strength was discovered for other ones [218]. This inconsistence is not surprising because the manufacturing methods used to produce test samples varied from one author to the other. Therefore, the only remaining fact is that the hardened formulations are brittle and hence worthless for load-bearing applications [4,5].

Setting process of the most types of apatite-forming formulations occurs according to just one chemical reaction [see chemical Equations (1) and (4)–(6)] and at near the physiological pH, which might additionally contribute to the high biocompatibility [165,166,167]. Namely, for the classical formulation by Brown and Chow, the transmission electron microscopy results suggested the process for early-stage apatite formation as follows: when TTCP and DCPA powders were mixed in an orthophosphate-containing solution, TTCP powder quickly dissolved due to its higher solubility in acidic media. Then the dissolved ions of calcium and orthophosphate, along with ions already existing in the solution, were precipitated predominantly onto the surface of DCPA particles. Few apatite crystals were observed on the surface of TTCP powder. At a later stage of the reaction, an extensive growth of apatite crystals or whiskers effectively linked DCPA particles together and bridged the larger TTCP particles causing the setting [242].

However, Norian SRS^®^ and Cementek^®^ (Table 3) were found to set according to two chemical reactions: precipitation of DCPD, followed by precipitation of either CDHA or carbonate apatite:

α-Ca_3_(PO_4_)_2_ + Ca(H_2_PO_4_)_2_·H_2_O + 7H_2_O = 4CaHPO_4_·2H_2_O (7)

5.2CaHPO_4_·2H_2_O + 3.6CaCO_3_ = Ca_8.8_(HPO_4_)_0.7_(PO_4_)_4.5_(CO_3_)_0.7_(OH)_1.3_ + 2.9CO_2_↑ + 12H_2_O (8)

The initial chemical reaction (7) was very fast and provoked DCPD formation and initial setting within seconds. The second step was slower: DCPD reacted completely within several hours with remaining α-Ca_3_(PO_4_)_2_ and CaCO_3_ forming carbonate apatite according to Equation (8). The latter step caused the hardening. A similar two-step hardening mechanism was established for a formulation consisting of MCPM and CaO: in the first step, during the mixing time, MCPM reacted with CaO immediately to give DCPD, which, in the second step, reacted more slowly with the remaining CaO to give CDHA [74].

In addition, the setting mechanism of an apatite-forming formulation was investigated in details for a three-component mixture of TTCP, β-TCP and MCPM dry powders in convenient proportions and with the overall atomic Ca/P ratio equal to 1.67. Two liquid phases in a raw were used to damp the cement powder, initially it was water + ethanol (ethanol was added to slow down the hardening) and afterwards H_3_PO_4_ and sodium glycerophosphate were added to water to prepare a reactive liquid [158]. At the very beginning, DCPD was found to form according to two chemical equations:

Ca(H_2_PO_4_)_2_·H_2_O + β-Ca_3_(PO_4_)_2_ + 7H_2_O = 4CaHPO_4_·2H_2_O (9)

Ca_4_(PO_4_)_2_O + 2H_3_PO_4_ + 7H_2_O = 4CaHPO_4_·2H_2_O (10)

The formation reactions of DCPD were fast and corresponded to the initial setting. Afterwards, TTCP reacted with the previously formed DCPD and with β-TCP to give CDHA according to the equations:

2Ca_4_(PO_4_)_2_O + 2CaHPO_4_·2H_2_O = Ca_10−*x*_(HPO_4_)*_x_*(PO_4_)_6−*x*_(OH)_2-*x*_ + *x*Ca(OH)_2_ + (4 – *x*)H_2_O (11)

2Ca_4_(PO_4_)_2_O + 4β-Ca_3_(PO_4_)_2_ + (2 + 2*x*)H_2_O = 2Ca_10-*x*_(HPO_4_)*_x_*(PO_4_)_6−*x*_(OH)_2−*x*_ + 2*x*Ca(OH)_2_ (12)

The formation reactions of the CDHA phase were quite slow and corresponded to the hardening stage. Although OCP was not detected in that study, its formation as an intermediate phase was postulated for this formulation [158]. A similar suggestion on the intermediate formation of OCP was made for the setting mechanism of Brown-Chow classical formulation [125,130]; however, reliable evidences for its presence are still lacking [196,243]. Strong experimental evidences of the existence of a transient OCP phase during setting were found in still another study; however, that system contained sodium silicates [41]. In all cases, OCP was suggested to appear as an intermediate because it was a faster forming phase than CDHA. This hypothesis is based upon the classical studies performed by Brown *et al*. [244,245,246], about the precursor phase formation during chemical crystallization of apatites in aqueous solutions.

Solubility of the hardened apatite-forming formulations in aqueous solutions is expected to be rather similar to that of bone mineral. This means that they are relatively insoluble at neutral pH and increasingly soluble as pH drops down; this is an important characteristic of normal bone mineral that facilitates controlled dissolution by osteoclasts [234].

To conclude this part, one should mention, that in 2000 the U.S. bone substitute market for Norian SRS^®^ accounted for ~15% of the total sales, followed by BoneSource^TM^ at ~13%, and α-BSM^®^ at ~8.5% [160].

### 3.2. Brushite-Forming Formulations

As indicated by its name, DCPD is the major product of the setting reaction for brushite-forming formulations [chemical Equations (2) and (3)], although traces of the un-reacted starting compounds can be present. Mirtchi and Lemaître [173] and independently Bajpai *et al*. [32] introduced this type of the cements in 1987. Up to now, several formulations have been already proposed, e.g., β-TCP + MCPM [173,174], β-TCP + H_3_PO_4_ [32,33,34] and TTCP + MCPM + CaO [247]. The full list of brushite-forming formulations is available in a topical review on the subject [248]. As seen from the chemical composition, all types of the brushite-forming formulations are set by the acid-base interaction only. As DCPD can only be precipitated at the solution pH < 6 (Table 1), the pastes of the self-setting brushite-forming formulations are always acidic during hardening [34,249]. For example, during setting of a β-TCP + MCPM formulation, the formulation pH varies from very acidic pH values of ~2.5, to almost neutral pH values of ~6.0 [34]. Replacing MCPM by H_3_PO_4_ renders the paste very acidic for the initial ~30 s but then the pH profile follows that obtained with MCPM. It is important to notice, that β-TCP + H_3_PO_4_ formulations have several advantages over β-TCP + MCPM ones, namely: (i) easier and faster preparation; (ii) a better control of the chemical composition and reactivity; (iii) improved physico-chemical properties, such as longer setting times and larger tensile strengths due to a higher homogeneity. However, the use of H_3_PO_4_ might impair the biocompatibility of the formulations, due to low pH values during setting [34].

As the solubility of calcium orthophosphates generally decreases with increasing of their basicity (Table 1 and Figure 1), the setting time of brushite-forming formulations much depends on the solubility of a basic phase: the higher its solubility, the faster the setting time. Therefore, the setting time of formulations made of MCPM + a basic calcium orthophosphate increases in the order: HA > β-TCP > α-TCP [4,5]. For example, HA + MCPM mixtures have a setting time of several minutes, β-TCP + MCPM mixtures—of 30 to 60 s and α-TCP + MCPM mixtures—of a few seconds [173,174]. Furthermore, if brushite-forming formulations contain an excess of a basic phase, the equilibrium pH will be given by the intersection of the solubility isotherms of the basic phase with that of DCPD. For example, the equilibrium pH values of β-TCP + MCPM, HA + MCPM and TTCP + MCPM mixtures were found to be 5.9, 4.2 and 7.6, respectively [4,5]. Follow-up of the chemical composition by ^31^P solid-state nuclear magnetic resonance (NMR) technique enabled to show that the chemical setting process for β-TCP + MCPM formulation reached the end after ~20 min [250]. Nevertheless, despite this initial high reactivity, the hardening reaction of brushite-forming formulations typically lasts one day until completion [235,236]. Additives that inhibit the crystal growth of DCPD have successfully been used to increase the setting time of β-TCP + MCPM mixtures [251]. Interestingly, contrary to apatite-forming formulations, the brushite-forming ones can be initially liquid and still set within a short period of time [4,5].

By itself, brushite is remarkably biocompatible and bioresorbable [249]. Due to both a better solubility of DCPD if compared to that of CDHA (Table 1 and Figure 1) and metastability of DCPD under physiological conditions [252], after implantation brushite-forming formulations are faster degradable than apatite-forming ones [253,254,255]. They are quickly resorbed *in vivo* and suffered from a rapid decrease in strength (although the mechanical properties of the healing bone increase as bone ingrowth occurs [51]). Short setting times, low mechanical strength and limited injectability seem to prevent brushite-forming formulations from a broader clinical application. However, the major reason why they are not more widespread is probably not related to the mechanical issues but just to a later arrival on the market. Use of sodium citrate or citric acid as setting retardants is an option to get more workable and less viscous pastes of brushite-forming formulations [38,256,257,258,259]. Similar effect might be achieved by addition of chondroitin 4-sulfate [260] and glycolic acid [261]. For the formulations with H_3_PO_4_ as the initial reactant [chemical Equation (3)], acid deficient formulations were also found to improve the workability. In this case, the setting reaction might be described by the following chemical equation [259]:

3.7β-Ca_3_(PO_4_)_2_ + H_3_PO_4_ + 27.8H_2_O = 3CaHPO_4_·2H_2_O + 2.7β-Ca_3_(PO_4_)_2_ + 21H_2_O (13)

Although several studies revealed that too much of DCPD in a given volume was not detrimental to the biological properties of brushite-forming formulations [51,234,247], occasionally, when large quantities of them were used, a certain degree of tissue inflammation during the first weeks of *in vivo* implantation were reported [255,259,262]. Further investigations indicated that the inflammatory could be due to a partial transformation of DCPD into CDHA with release of orthophosphoric acid [263]:

(10 − *x*)CaHPO_4_·2H_2_O = Ca_10−*x*_(HPO_4_)*_x_*(PO_4_)_6−*x*_(OH)_2−*x*_ + (4 – *x*)H_3_PO_4_ + (18 – *x*)H_2_O (14)

Transformation of DCPD into CDHA occurs via two successive processes: dissolution and precipitation [264] and can be retarded by adding magnesium ions to the formulations, thus reducing the possibility of inflammation [4,5]. The aforementioned case of acid deficient formulations [chemical Equation (13)] is the second option, because it reduces the amount of un-reacted acid [259] with an option to consume liberating in chemical Equation (14) H_3_PO_4_ by the excess of β-TCP. Implantation of previously set brushite-forming formulations might be the third option, because a solid bioceramics was found to be better tolerated than paste implants. Besides, more bone was formed at the solid implant contact and the solid material degraded not so rapidly [265]. For the hardened brushite formulations, a linear degradation rate of 0.25 mm/week was reported [266]. This rapid degradation rate might lead to formation of an immature bone. Adding β-TCP granules to the self-setting pastes could solve this problem because the granules might act as bone anchors and encourage formation of a mature bone [266,267].

To finalize this topic, one should briefly mention on a possibility to precipitate DCPA (monetite) instead of DCPD. It is well known, that DCPA might be crystallized under the same conditions as DCPD but either from aqueous solutions at elevated (>~90 °C) temperatures or at ambient conditions but in water-deficient environments [249,268]. Therefore, monetite-forming self-setting calcium orthophosphate formulations could exist. Indeed, there are several publications, in which formation of monetite instead of brushite has been detected as the final product [29,35,269,270,271,272]. In addition, monetite might be formed during a prolonged storage of dry powders of brushite-forming formulations in normal laboratory atmosphere (~60% relative humidity) [273]. Therefore, one might claim on an incipient topic of self-setting monetite-forming calcium orthophosphate formulations, which has a potential to be developed in a league of its own.

Additional details on the self-setting brushite-forming formulations might be found in a recent review on the subject [248].

## 4. Various Properties

### 4.1. Setting and Hardening

Generally, self-setting calcium orthophosphate formulations must set slowly enough to provide sufficient time to a surgeon to perform implantation but fast enough to prevent delaying the operation. Ideally, good mechanical properties should be reached within minutes after initial setting. Two main experimental approaches are used to study the setting process: a batch approach and a continuous approach. In the batch approach, the setting reaction is stopped at various times and the resulting samples are analyzed to determine, e.g., the composition and compressive strength of the samples [235,236]. There are currently two standardized methods to apply this approach, namely, Gillmore needles method (ASTM C266-89) [274] and Vicat needle method (ASTM C191-92) [275]. The idea of both methods is to examine visually the sample surfaces to decide whether the formulation has already set, *i.e*., if no mark can be seen on the surface after indentation. Besides, the setting process might be monitored in real time by non-destructive methods (the continuous approach), e.g., using pulse-echo ultrasound technique [276,277], isothermal differential scanning calorimetry [198,199,278,279,280,281,282,283,284] and alternating current (AC) impedance spectroscopy [285]. For example, calorimetry measurements suggested that in Equation (2) the endothermic MCPM dissolution and the highly exothermic β-TCP dissolution occurred simultaneously, followed by the exothermic crystallization of DCPD [282]. Thus, brushite-forming formulations usually warm upon final setting [278]. Moreover, acid-base reactions (1)–(3) can be and have been analyzed by measuring the pH evolution of diluted pastes [235]. In addition, non-destructive methods of Fourier-transform infrared spectroscopy [40,41,43,283,286], solid state NMR [250], X-ray diffraction [40,43,66,175,287] and energy dispersive X-ray diffraction [40,41,42,43,288,289,290] might be applied as well. The latter techniques proved to be powerful even though they have limitations such as the time required for each measurement (250 s for an X-ray diffraction scan is a problem for fast setting reactions). In addition, the analysis is often located at the sample surfaces where evaporation and thermal effects can modify the reaction rates if compared to those in the bulk. Furthermore, the continuous approaches are indirect, which markedly complicates an interpretation of the collected data, particularly in complex formulations [235].

**Figure 6:**
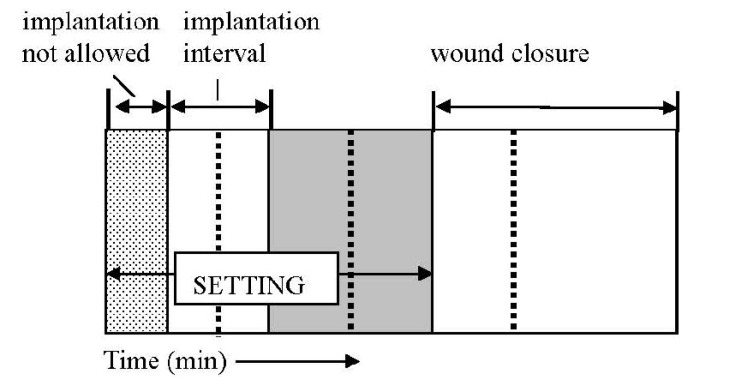
A diagram of the setting parameters relevant for a self-setting calcium orthophosphate formulation: *CT*—cohesion time; *I*—initial setting time; *F*—final setting time. Adapted from [46] with permission.

A way to assess the hardening kinetics is to measure its setting time, which means the time required to reach a certain compressive strength, generally close to 1 MPa. The most straightforward approach is to prepare self-setting samples with a well-controlled geometry (e.g., cylinders), incubating those samples for various times in the right environment (temperature, humidity) and assessing the composition and mechanical properties of the samples as a function of time [235]. One should stress, that setting time for calcium orthophosphate formulations often corresponds to an earlier stage in the overall setting reaction, typically 5%–15% of the overall reaction, while the end of the hardening process is typically reached after several days [130,196]. Gillmore needles have been used with success to measure the initial (*I*) and final (*F*) setting times of calcium orthophosphate cements [123]. Namely, a light and thick needle is used to measure the initial setting time *I*, while a heavy and thin needle for the final setting time *F* [153]. The clinical meaning is that the cement paste should be implanted before time *I* and that the wound can be closed after time *F* (Figure 6).

The implanted formulations should not be deformed between times *I* and *F* because in that stage of the setting any deformation could induce cracks [46]. The following handling requirements have been formulated for calcium orthophosphate cements, as a result [153,291]:

3 min ≤ *I* < 8 min

*I* − *CT* ≥ 1 min

*F* ≤ 15 min

These parameters are represented schematically in Figure 6. The second requirement means that the cohesion time (*CT*) must be at least 1 min before *I*, so that a clinician has at least 1 min to apply and to mold the material. *CT* is the time from which a formulation no longer disintegrates when immersed in Ringer’s solution [153]. As the mixing in a mortar is about 1 min, the shortest *CT* that can be allowed is about 2 min, so that a clinician has at least 1 min to collect the paste from the mortar and put it on a pallet knife or into a syringe with which it is to be transferred to the wound after *CT* and before *I* [153]. For dental applications, time *I* must be close to 3 min, whereas for orthopedic applications it must be close to 8 min. However, in no case it will be tolerable for the clinicians if time *F* becomes greater than 15 min [46,153].

### 4.2. Phase Mixing

In the clinical situation, self-setting calcium orthophosphate formulations can be either applied by the fingertips of a surgeon or injected from a syringe to the defect area of a bone. The first type of the application requires formulation of high-viscous self-setting pastes and putties, which can be applied manually as dough, while the second type requires formulation of low-viscosity compositions, which can be applied by injection from a syringe [153]. Currently, injection appears to be the preferred method between these two major options. Thus, a compromise must be found between a high viscosity leading to too high injection forces and a low viscosity increasing the risk of extravasations. Thus, viscosity values in the range of 100–2000 Pa·s are generally considered to be adequate [292].

**Figure 7:**
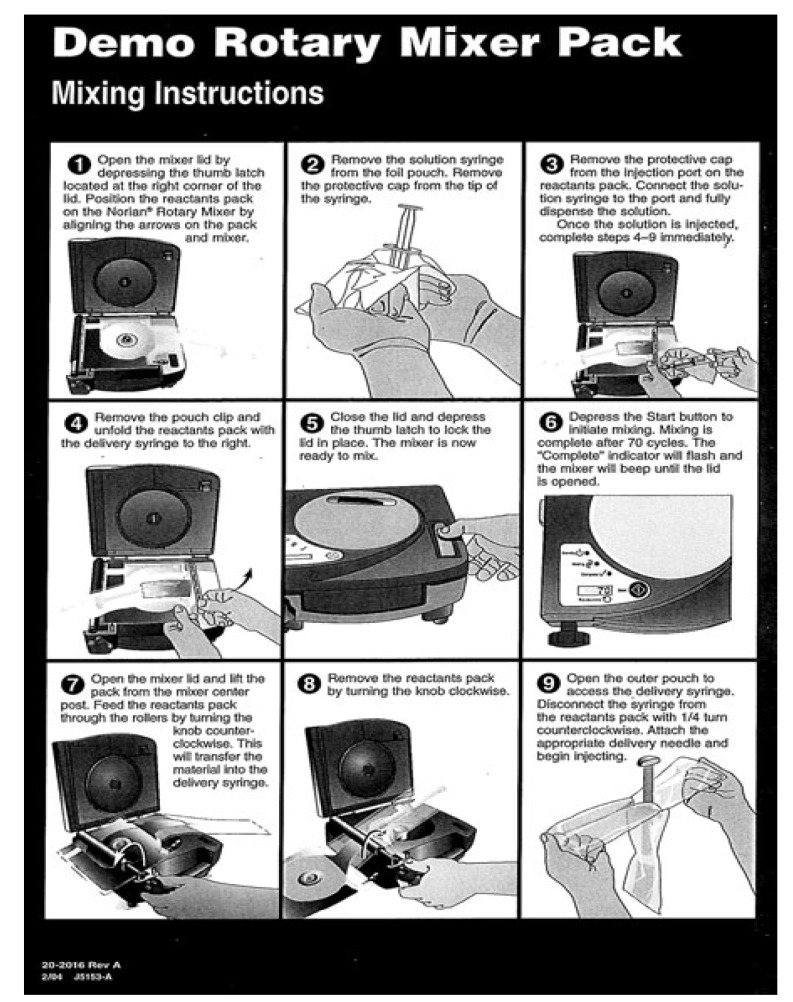
Mixing instructions for a Norian rotary mixer.

In any case, before using a surgeon needs to have a powder and a liquid be mixed properly and thoroughly (to avoid the powder/liquid encapsulation) within the prescribed time. This process must be performed in a sterile environment. Therefore, a mixing procedure is very important because prior to be injected, a self-setting paste must be transferred from a mixing chamber into a syringe. Ideally, this should be done without trapping air bubbles by the formulation [293]. Earlier, most calcium orthophosphate formulations were manually mixed with aqueous solutions using a mortar and either a pestle or a spatula. That time, some concerns were raised about an insufficient and inhomogeneous mixing thus compromising the implant strength, as well as on inconsistencies between operators causing unpredictable variations in graft performance [294]. Mechanical mixing (e.g., by either an electrically powered mixing machine of Norian SRS/CRS^®^ (Figure 7) or Mini-malax^®^ mixing system for Cementek^®^, produced by Teknimed S.A., City, Country) is the modern approach. It allows mixing the pastes within 60–80 s and enables a rapid and reliable filling of the application syringe [230]. Besides, a powder and a solution might be placed into a syringe and mixed inside a shaker to produce a consistent self-setting paste of the desired viscosity [293]. A mechanical mixing was found to decrease both the mean viscosity of the curing pastes and variability in the viscosity at a given time [295]. However, it did not improve the mechanical strength of the hardened formulations [4,5].

Of the commercial formulations, listed in Table 2, Norian SRS^®^ is sold as a reactant pack containing two components: a mixture of dry powders (MCPM + α-TCP + CaCO_3_) and a liquid (aqueous solution of Na_2_HPO_4_). The components are mixed in the operating room. The paste that is formed is malleable and injectable for ~5 min; it hardens within ~10 min after injection [22,234]. However, data are available that out of 4.5 mL Norian SRS^®^ cement paste ~3 mL is injectable only, whereas up to 1.5 mL of the paste might remain uninjectable from the syringe [46]. This phenomenon is prescribed to the formulation rheology and its interaction with the hydraulic forces of the syringe. α-BSM^®^ (Table 2) is also a two-component system; it is prepared from a mixture of ACP and DCPD powders and a saline solution [193]. Biopex^®^ consists of four different calcium orthophosphates: 75 wt% α-TCP, 18 wt% TTCP, 5 wt% DCPD and 2 wt% HA (Table 3). The aqueous solution contains 12 wt% sodium succinate and 5 wt% sodium chondroitin sulfate [296]. Effects of liquid phase on the basic properties of Biopex^®^ were investigated. When mixed with neutral sodium hydrogen orthophosphate or succinic acid disodium salt solution, the initial setting times of the cement were 19.4 ± 0.55 and 11.8 ± 0.45 min, respectively. These setting times were much shorter than that of distilled water, 88.4 ± 0.55 min [297]. Biopex^®^ is mixed with a spatula inside a syringe that can be opened from the front. After mixing, the front part is closed, a needle is inserted into this front part and the cement paste can be manually injected [4,5].

Several systematic studies on the influence of composition and concentration of the liquids used in preparing of self-setting calcium orthophosphate formulations were performed as well [38,256]. Unfortunately, the results appeared to be rather unclear. For example, for several formulations, mixing with sodium citrate or citric acid resulted in some effects on the initial setting time [38,257], while for other ones the effect was insignificant [256]. Concentration increasing of sodium citrate solution resulted in initial setting time increasing [38,256], although the injectability variations of the cement pastes were inconsistent [38,257].

### 4.3. Rheological Properties

**Figure 8:**
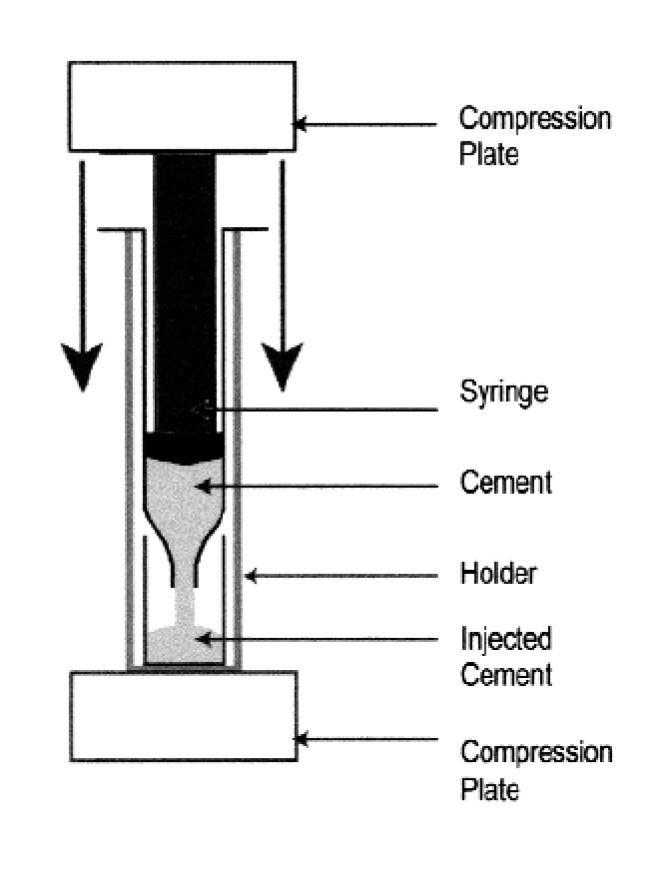
A schematic representation of the experimental setup used to quantify the injectability of the calcium orthophosphate formulations. Reprinted from [305] with permission.

In terms of the rheological properties, all types of self-setting calcium orthophosphate formulations belong to non-Newtonian fluids. The latter means that the viscosity of such fluids is dependent on shear rate or shear rate history. Nevertheless, good injectability, adequate viscosity and satisfactory cohesion are required for the successful biomedical applications [298,299]. Among them, injectability is defined as an ability of a formulation to be extruded through a small hole of a long needle (e.g., 2 mm diameter and 10 cm length) [300,301] (other needles are also applied [302,303]); and for certain applications, injectability is even a prerequisite. It is measured by the weight percentage of the formulation that could be injected without demixing from a standard syringe by either a hand or a force of 100 N maximum (Figure 8). The numerical values are calculated by the following equation [304]:

*Inj* = (*W_F_* − *W_A_*)/(*W_F_* − *W_E_*) × 100%

where *Inj* is the percentage injectability; *W_E_* is the weight of the empty syringe; *W_F_* is the weight of the full syringe and *W_A_* is the weight of the syringe after the injection.

Usually, injectability of calcium orthophosphate formulations are varied inversely with their viscosity, the P/L ratio, as well as the time after starting the mixing of liquid and powder [72,301,305]. In addition, powder reactivity was shown to influence the injectability. Namely, significant differences were observed between the injection behaviors of the non-hardening β-TCP pastes and self-hardening α-TCP pastes, α-TCP being less injectable than β-TCP and requiring higher injection loads. What is more, the parameters affecting powder reactivity were shown also to affect injectability. Thus, whereas powder calcination resulted in increased injectability, an addition of setting accelerants tended to reduce the injectability [304]. Furthermore, injectability is improved with smaller particle sizes, with shorter and larger diameter cannula, as well as at smaller flow rates [300]. Moreover, particle shape of the powder is also expected to have effects on the injectability. Namely, powders with spherical shape or round particles are easy to roll and thus good handling properties and injectability are found when pastes are prepared from such materials. Besides, it should be noted that the paste could become fluid with less amount of liquid phase since no captured liquid exists in the case of spherical powder [306].

Unfortunately, when a self-setting formulation, which is a biphasic mixture of a finely divided ceramic (powder, granules) and a liquid, is submitted to a pressure gradient, the liquid may flow faster than the solid, resulting in local changes of the paste composition. Specifically, the paste present in the region of the highest pressure (e.g., close to the plunger of a syringe) may become so depleted in liquid that the biphasic mixture in this zone is not longer a paste but a wet powder [300,302]. Contrarily, the paste in the zone of the lowest pressure (e.g., at the cannula tip) is enriched in liquid. Since these effects are dynamic, the size of the zone depleted in liquid (wet powder) increases during injection, eventually reaching the tip of the injection device and plugging it. The phenomenon, in which the pressure applied to the paste provokes a phase separation after a certain injection time, is generally referred as filter pressing, phase separation or phase migration [149] (see the aforementioned example for Norian SRS^®^ [46], in which a thick mass remained inside a syringe).

Possible mechanisms underlying the limited injectability of self-setting calcium orthophosphate formulations have been discussed in literature [303,307]. In the case of demixing, the exact composition of the extruded part of the paste becomes unknown. Moreover, due to a deviation from the initial P/L ratio, it becomes unclear whether the setting behavior and the mechanical and histological properties of the extruded part are still clinically acceptable. Therefore, a good cohesion of the paste is necessary in order to avoid these problems [308].

Cohesion (= cohesiveness, “non-decay”) is the ability of a paste to keep its geometrical integrity in an aqueous solution [149]. It is evaluated by measuring the amount of solid particles released from the formulation prior to its final setting. For self-setting formulations, a bad cohesion may prevent setting and may lead to negative *in vivo* reactions due to the release of microparticles [309]. Since a high cohesion is the result of strong attractive forces among the particles, factors enhancing van der Waals forces (attractive) and decreasing electrostatic forces (repulsive) can be used to improve cohesion [149]. For example, an appropriate cohesion was achieved when no disintegration of the paste was observed in the fluid [153,308]. This can be accomplished by keeping a high viscosity for self-setting pastes [22] or using cohesion promoters (e.g., 1% aqueous solution of sodium alginate [200,310,311], as well as other chemicals [200,312,313,314]). Some calcium orthophosphate formulations fulfill both criteria, e.g., Norian SRS^®^, but others fulfill only one or even none of these requirements. For example, BoneSource^TM^ [127] and Cementek^®^ (Table 3) are not injectable and blood must be kept away from the implanting site until setting [4,5]. A poor cohesion has been associated to a poor biocompatibility that might lead to inflammatory reactions [309]. Further details on the cohesion properties of various calcium orthophosphate pastes are available in literature [308].

Viscosity is a measure of the resistance of a fluid, which is being deformed by either shear stress or tensile stress. Generally, the viscosity in the range of 100–1000 Pa·s appears to be ideal [315] and, if possible, a self-setting formulation should have a constant viscosity in the indicated range. Unfortunately, viscosity of self-setting formulations is not a constant value, which, after a decrease in the first seconds after mixing, increases considerably during curing, eventually leading to hardening. Furthermore, viscosity should be high enough to prevent extravasation; therefore, it is very important to define an adequate injection window [315].

### 4.4. Properties Improving

As written above, the properties of the existing self-setting calcium orthophosphate formulations are not ideal. Several ways can be adopted to improve them. The first approach consists of injectability improvement. There are several options for this. Firstly, the injection device can be modified. For example, shorter cannulas with a larger diameter, as well as smaller injection rates favor a good injectability. The last option is not so straightforward: for example, Habib *et al*. have shown that large injection rates are not detrimental to injectability because of the shear-thinning behavior of many calcium orthophosphate cements [303]; Secondly, an external energy might be applied. For example, injectability was improved by ultrasonication, which was believed to result from a reduction in the injection force *versus* the filtration force as a result of a lesser reduction in the particle interaction and the paste flowability [316]; Thirdly, the formulation composition can also be adapted. Namely, a decrease of the particle size, the P/L ratio and the plastic limit was found to contribute to a better injectability [300,305]. For example, injectability was found to be unaffected by P/L ratio within the range of 3.85–4.50 g/mL but drops by nearly 100% between P/L ratio of 4.50 and 5.00 g/mL [38]. However, a decrease in P/L ratio leads to a decrease in the mechanical properties of the self-setting formulations and cohesion might be destroyed. Furthermore, both the initial and final setting times decreased markedly with the P/L ratio increasing [256,317]. Therefore, variations in the P/L ratio appear to be valid to a certain extent only. That is why the manufacturer of Biopex^®^ suggests using a P/L ratio of 2.8 or 3.3 g/mL.

Particle size decreasing of calcium orthophosphate crystals is the second approach for the injectability improvement. For example, α-BSM^®^ is well injectable because it consists of small crystals. Even though small particles require a larger amount of mixing liquid to obtain a paste, injectability and cohesion of such formulations are generally very good [4,5]. An indirect approach is to add calcium orthophosphate crystals those act as spacers between other particles. For example, DCPA is added to the formulation of Biocement D^®^ to improve injectability [4,5]. Similarly, there is an apatite-forming formulation containing spherical particles of TTCP to improve injectability [318].

Using various chemical additives is the second way to improve the properties of the self-setting formulations [319]. For example, water demand of can be reduced by ionically modifying the liquid component, e.g., by adding nontoxic sodium salts of α-hydroxy di- and tri- acids [320,321]. A list of additives, that have been already studied, includes fluidificants, air-entraining agents, porogens, workability-improvement agents, setting time controllers and reinforcing additives [182,231,322]. Besides, various radiopacifiers might be used to simplify an un-invasive *in vivo* monitoring of the implanted cements [323,324,325,326,327]. The main role of fluidificants is to reduce a mixing time of the formulations. Citric acid is an example of this reagent; it retards the dissolution-precipitation reactions, decreases the compressive strength during initial setting but increases its strength in the final stages of hardening [257]. Furthermore, data are available, that citric acid decreases the setting time and improves the mechanical properties of the hardened formulations [328]. Adding of surfactants to the self-setting formulations was found to have two different meanings: they might act as both air-entraining agents by lowering the surface tension [329,330] and interaction modifiers by shifting the isoelectric point [331].

In addition, studies are available, in which self-setting calcium orthophosphate formulations were modified by various bioorganic compounds in attempts to influence the bone healing process [332,333,334,335]. For example, there is a study, in which a self-setting formulation was set in the presence of cocarboxylase, glucuronic acid, tartaric acid, α-glucose-1-phosphate, l-arginine, l-aspartic acid and l-lysine, respectively, with the aim to influence formation and growth of CDHA crystals through the functional groups of these biomolecules [335]. Except for glucuronic acid, all these modifications were found to result in the formation of smaller and more agglomerated CDHA particles, which had a positive impact on the biological performance indicated by first experiments with the human osteoblast cell line hFOB 1.19. Moreover, initial adhesion of human bone marrow-derived mesenchymal stem cells was improved on the formulations containing cocarboxylase, arginine and aspartic acid. Furthermore, cell proliferation was enhanced on the formulations modified with cocarboxylase and arginine whereas osteogenic differentiation remained unaffected. Besides, the formulations with arginine and aspartic acid, but not with cocarboxylase, led to a higher BMP-2 binding [335].

Since a good adhesion to bones and other structures allows better transmission of forces at the formulation-bone interfaces, a proper adhesion between the set formulations and bones is very important for many surgical procedures. Chemical additives might also improve adhesive properties of self-setting calcium orthophosphate formulations. For example, it was observed that brushite-forming formulations set with pyrophosphoric acid in the liquid phase had an increased adherence to various surfaces such as bone, alumina, sintered HA and stainless steel [336].

Porosity is a very important property to provide good *in vivo* bioresorption of implanted biomaterials. Thus, various air-entraining agents and porogens are commonly used to induce macroporosity of self-setting calcium orthophosphate formulations, ideally, without affecting their normal setting. For example, crystals of mannitol, CH_2_OH(CHOH)_4_CH_2_OH, were tested as an air-entraining agent; however, both loss of workability during mixing and severe depreciation of mechanical properties were discovered simultaneously [337,338,339,340,341,342]. Other porogenic agents were also tested to create porosity. The examples include: hydrogen peroxide in the liquid phase [343] and/or iced [344], crystals of NaHCO_3_ and Na_2_HPO_4_ [345], calcium sulfate [58], calcite [247] and NaCl [346,347], poly(d,l-lactic-co-glycolic acid) microparticles [348,349,350,351,352,353,354,355], microspheres of pectin [356], and gelatin [357,358], vesicants [359], cetyltrimethyl ammonium bromide [360], polytrimethylene carbonate [361], sucrose granules, as well as some immiscible liquids. These additives could be applied on pre-set formulations only, while the solubility degree of the solid porogens during setting influences both the content and dimensions of the macroporosity. After hardening, dissolution of the remaining soluble porogens in either water or body fluids produces macropores with the dimensions and shapes of the dissolved crystals. One important limitation that can be envisaged from this route is the need to add a large amount of porogenic agents to guarantee pore interconnectivity, thus compromising not only the excellent biocompatibility and bioactivity of self-setting calcium orthophosphate formulations but also their injectability. Another shortcoming is a lack of strength of the resulting bioceramics, especially if particulates dissolve quickly, greatly limiting its applications. An innovative approach that aims at overcoming the lack of interconnectivity and initial strength consists in using resorbable fibers [362,363,364,365,366,367,368,369,370]. These fibers have the function of initial reinforcing, providing the needed short-term strength and toughness, and gradually dissolving afterwards, leaving behind macropores suitable for bone ingrowth. One interesting advantage of long fibers over particulates and short fibers is the fact that once resorbed they can form interconnected pores inside the solid structure facilitating bone tissue regeneration [371].

**Figure 9:**
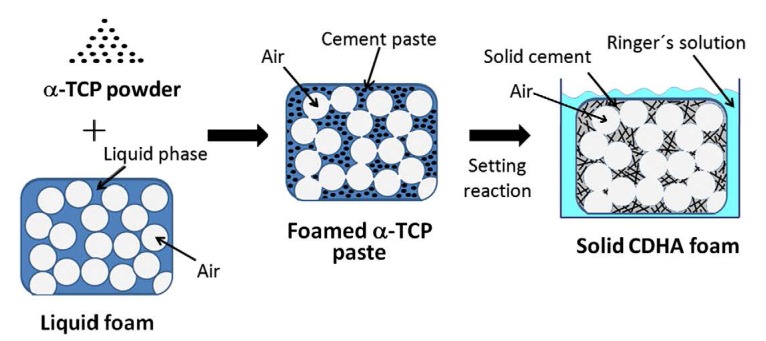
A schematic drawing of calcium-deficient hydroxyapatite (CDHA) foams preparation. Initially a liquid was formed by mechanical agitation of an aqueous solution of a soluble surfactant. Then, the foam was mixed with α-TCP powder, producing a foamed paste, which was either cast or directly injected into the moulds. The setting reaction produced hydrolysis of α-TCP to CDHA, which resulted in foam hardening. Reprinted from [378] with permission.

One more approach to create porosity consists in adding solid NaHCO_3_ to the starting powder and using two different liquids: first, a basic liquid to form the paste, and later an acid liquid to obtain CO_2_ bubbles to create porosity [372]. Besides, pore forming CO_2_ bubbles appear at hardening of apatite-forming formulations, consisting of an acidic calcium orthophosphate, such as MCPM or DCPD, and either CaCO_3_ [30,50,69,70,71] or NaHCO_3_ [373,374,375]. Furthermore, addition of an effervescent porogen formulation comprised from NaHCO_3_ (54.52%) and citric acid monohydrate (45.48%) has been suggested [376]. More to the point, the liquid phase of a formulation might be initially foamed and subsequently mixed with the self-setting powders. In this case, the setting reactions transform the liquid foam into a solid, which ideally maintains the geometry, size and shape of the bubbles (Figure 9). Thus, the liquid foam acts as a template for the macroporosity of the solid foam [343,377,378,379]. In addition, several other porosity creation techniques for self-setting calcium orthophosphate formulations are known and, for further details on the subject, the interested readers are referred to an excellent review [371].

The major examples of workability-improvement agents, which are added to the self-setting formulations, include water-soluble polymers. Specifically, polysaccharides [120,132,380,381,382,383], gelatin [317,384,385,386,387,388,389,390] and polyacrylic acid [391,392,393] are of an interest due to their biocompatibility and good rheological properties. Only small amounts (a few weight %) are needed to dramatically increase the viscosity of the pastes. Besides, the pastes become more cohesive and highly resistant to washout immediately after mixing. For example, a 5 wt% sodium chondroitin sulfate solution is used as mixing liquid in Biopex^®^ [4,5]. In the case of gelatin, more than a 50% improvement of the compressive strength was detected [386]. The gelatin-containing formulations after setting were found to exhibit reduced crystallinity, much smaller CDHA crystals and a more compact microstructure; all these phenomena might be accounted for the improved mechanical properties [387]. In addition, the presence of gelatin improved mechanical properties of the formulations; in particular, the formulations containing 2 wt% gelatin were found to harden in an acceptable time and were recommended for clinical applications [390]. In some cases addition of a gelling agent might cause an increase in hardening time [394] but this was remedied by the use of a sodium orthophosphate solution as the liquid phase [166,167]. Most polysaccharide solutions are thixotropic, *i.e*., the viscosity of the solution decreases as the shear rate increases. Certain polysaccharides, such as sodium alginate, pectize in contact with calcium ions. This property can be used to make putty-like cement pastes [22]. However, only few polysaccharides are accepted for parenteral use [4,5]. Nevertheless, the use of gelling agents widened a possible application of the self-setting calcium orthophosphate formulations because such formulations can be used even when complete homeostasis is difficult.

Of two families of the self-setting formulations, brushite-forming ones react generally much faster than apatite ones. As a result, to satisfy the clinical requirements (Figure 6), setting time of brushite-forming formulations has to be prolonged, whereas that of apatite-forming ones has to be shortened [4,5]. According to the aforementioned, setting reactions of any self-setting calcium orthophosphate formulation consists of three successive stages: (1) dissolution of reactants to saturate the mixing liquids by calcium and orthophosphate ions; (2) nucleation of crystals from the supersaturated solutions; (3) growth of crystals. Therefore, experimental approaches to modify the setting kinetics are to be targeted to these three stages. The available approaches have been summarized in Table 4 [235]. Furthermore, seven strategies have been described to decrease the setting time of calcium orthophosphate formulations [236]. They are: (i) mean particle size decreasing of the initial powders; (ii) the P/L ratio increasing; (iii) pH drop of the mixing liquid to increase calcium orthophosphate solubility and hence accelerate the chemical transformations; (iv) a nucleating phase addition, such as a nano-sized HA powder; (v) adding orthophosphate and/or calcium ions into the mixing liquid to accelerate the setting reaction according to the common-ion effect; (vi) solubility reducing of the reaction end-product, for example, by adding fluoride ions into the mixing liquid; (vii) solubility increasing of the starting material by amorphization, e.g., by prolonged milling. For further details on these strategies and approaches, as well as for application examples, the interested readers are referred to the original publications [235,236].

Various setting time controllers (accelerators and retardants) are used to influence the setting time. They include sodium hydrogen pyrophosphate (Na_2_H_2_P_2_O_7_) and magnesium sulfate (according to other studies, ions of citrate, sulfate and pyrophosphate are necessary [251,395]), which are added in amounts <1 wt% [396]. Application of biocompatible α-hydroxylated organic acids (glycolic, lactic, malic, tartaric and citric acids) and their calcium and sodium salts for modification of both rheological and setting properties of calcium orthophosphate formulations is well described elsewhere [397,398]. Besides, aqueous solutions of sodium orthophosphates [120,239,273,353,399,400,401] and gelatinized starch [402] are also known as setting time accelerators. An extensive list of the compounds, which might be suitable as accelerators, retarders, additives or reactants in calcium orthophosphate cement formulations, might be found in literature [123]. Interestingly that in some cases a simple thermal treatment of the initial reagents (in that particular case, α-TCP powder) at ~500 °C could extend the initial part of the setting reaction from a few minutes to a few hours hence providing a potential approach to better control the setting process [403,404].

**Table 4:** List of strategies and approaches to modify reactivity of the self-setting calcium orthophosphate formulations [235].

Strategy	Approach	Sub-approaches
1. Dissolution rate	1.1. Change contact area between reagent and mixing liquid	1.1.1. Change milling duration
		1.1.2. Use nano- or micron-sized powders
	1.2. Change solubility in the mixing liquid	1.2.1. Use more/less soluble phase
		1.2.2. Change of reaction pH
	1.3. Change saturation of the mixing liquid	
	1.4. Use dissolution inhibitors in the mixing liquid	
	1.5. Modify reagent surface	1.5.1. Chemical change (pre-reaction)
		1.5.2. Physical change (dissolution pits)
2. Nucleation rate	2.1. Use crystallization nuclei	
	2.2. Change the saturation of the reaction product in the mixing liquid	2.2.1. Change of saturation
		2.2.2. Change of end-product solubility
	2.3. Use nucleation inhibitors	
3. Growth rate	3.1. Change the saturation of the reaction product in the mixing liquid	3.1.1. Change of saturation
		3.1.2. Change of end-product solubility
	3.2. Use crystal growth inhibitors	

The subject of the reinforcing additives is discussed in details below in Section 7.

Concerning storage stability and shelf life, the factors, significantly influencing those properties for the initial dry powders of calcium orthophosphate formulations, were found to be temperature, humidity and a mixing regime of the powders. Various storage conditions appeared to be effective in prolonging the stability of dry brushite-forming formulations. In the order of effectiveness, they were ranged: adding solid citric acid retardant > dry argon atmosphere ≈ gentle mixing (minimal mechanical energy input) >> low temperature [273]. Finally, the self-setting formulations must be sterilized before a clinical use. A detailed description of the sterilization techniques might be found elsewhere [405].

## 5. Bioresorption and Replacement of the Self-Setting Formulations by Bones

Due to the excellent bioresorbability of DCPD and CDHA, a newly forming woven bone might substitute the hardened calcium orthophosphate formulations. Namely, the implants made of hardened BoneSource^TM^ (an apatite-forming formulation) were found to be partly resorbed and replaced by natural bone, depending upon the size of the cranial defect [127]. Replacement of BoneSource^TM^ by bone with a minimal invasion of connective tissue was detected in another study, while ChronOS^TM^ Inject (a brushite-forming formulation) samples exhibited a higher rate of connective tissue formation and an insufficient osseointegration [406]. α-BSM^®^ was evaluated in a canine femoral slot model. New bone was found to form in 3 weeks via an osteoconductive pathway. After 4 weeks, only ~1.7% of the implanted material was observed. The hybrid bone possessed the strength of normal, unoperated bone after 12 weeks. In 26 weeks, the boundary between old and new bones was virtually indistinguishable, with only ~0.36% of the implant recognizable [193]. Neither influence on general health, limb specific function and pain, nor associated complications with α-BSM^®^ application were found past 2 years in another study [407]. Norian SRS^®^ was evaluated in canine tibial and femoral metaphyseal defects. The hardened formulation appeared to be gradually remodeled over time, with blood vessels penetrating through it. However, some amounts of Norian SRS^®^ were detected in the medullary area as long as 78 weeks after being implanted in dog femurs [49]. An interesting study on the *in vitro* resorption of three apatite-forming formulations (conventional, fast-setting and anti-washout) by osteoclasts if compared with a similar resorption of sintered HA and a cortical bone revealed an intermediate behavior of the formulations: they were resorbed slower than bone but faster than HA [408]. Furthermore, bone neo-formation was seen 7 seven days after implantation of a self-setting α-TCP formulation [409]. The biodegradation rate of the formulations might be influenced by ionic substitutions in calcium orthophosphates [410]. Evidences of the direct contact of bone and a hardened calcium orthophosphate formulation without soft tissue interposition might be found in literature [411,412].

Different studies reported on both bioresorption and the progress of bone formation around hardened calcium orthophosphate formulations which in certain cases demonstrated both osteoconductive and osteoinductive properties [413]. However, there are studies in which the osteoinductive properties of self-setting calcium orthophosphate formulations were not confirmed [414]. Besides, inflammatory reactions were noticed when the formulation did not set [309]. Since the solubility of a non-stoichiometric CDHA is higher than that of stoichiometric HA, α- and β-TCP (Table 1), while the particle dimensions of a precipitated CDHA is smaller than that of sintered calcium orthophosphates, the biodegradability of apatite-forming formulations is always better than that of dense bioceramics made of sintered stoichiometric calcium orthophosphates. For example, histologically, at 2 weeks, spicules of living bone with normal bone marrow and osteocytes in lacunae could be seen in implanted formulations. At 8 weeks, the formulation was almost totally surrounded by mature bone. At this stage, no resorption was observed [415]. Only ~30% decrease of the implanted amount of Norian SRS^®^ was reported after 24 months in a rabbit femur [416]. Moreover, several differences could be expected depending on the formulation type. For example, as the product of BoneSource^TM^ and Cementek^®^ is a crystalline CDHA, both commercial formulations are expected to resorb slower than other apatite-forming formulations. Indeed no resorption of BoneSource^TM^ was observed after several years implantation; though some resorption of Biobone^®^ was detected. However, porosity appears to be the main biodegradability factor at play: the more porous (for cells) hardened formulation degrades faster than the less porous one [417]. For example, as Biobone^®^ is more porous than BoneSource^TM^, the discovered diversity could be due to the differences in porosity [4,5]. The latter conclusion is confirmed by the results of other studies: a positive influence of the porosity on resorption rates was found [311]. The interested readers are referred to a study on the suitability of hardened and porous calcium orthophosphate formulations as scaffolds for bone regeneration, using a rabbit model [418].

The bioresorption properties of bioceramics are generally believed to relate to the solubility of their constitutive phases. The implanted calcium orthophosphates might be bioresorbed by two possible mechanisms, namely: an active resorption, mediated by the cellular activity of macrophages, osteoclasts and other types of living cells (so called phagocytosis or literally “cell-eating”) [419,420,421] and a passive resorption due to either dissolution [6] or chemical hydrolysis (brushite-forming formulations only) [180,259] in the body fluids. Dissolution might be both chemical and physical. The former occurs with calcium orthophosphates of a low solubility (those with Ca/P ratio > ~1.3) in acidic environments, while the latter occurs with calcium orthophosphates of a high solubility (those with Ca/P ratio < ~1.3). For example, for MCPM, MCPA, DCPD and DCPA the solubility product are several times higher than the corresponding ion concentrations in the surrounding body fluids; therefore, they might be physically dissolved *in vivo*, which is not the case for α-TCP, β-TCP, CDHA, HA, FA, OA and TTCP since the surrounding body fluids are already supersaturated with regard to these compounds. Therefore, biodegradation of the latter materials is only possible by osteoclastic bone remodeling and is limited to surface degradation since cells cannot penetrate the microporous ceramic structure. Osteoclastic cells resorb calcium orthophosphates with Ca/P ratio > ~1.3 by providing a local acidic environment which results in chemical dissolution. In order to investigate two bioresorption mechanisms separately, experiments should be performed by incubating the samples in a cell culture medium without cells to study the passive resorption, whereas the active resorption should be determined during cell culturing on the sample surfaces [422]. Unfortunately, the factors concerning the biodegradation of calcium orthophosphate biomaterials have not been completely elucidated yet. The chemical composition, physical characteristics and crystal structures certainly play an important role in their biological behavior. In addition, biodegradation may be influenced by the investigational conditions, such as experimental models, implantation sites and animal species [420].

The data are available that macrophages and giant cells decompose quickly resorbed calcium orthophosphates (e.g., brushite-forming formulations) [255], while slowly (from months to years) resorbed apatite-forming formulations are decomposed by osteoclast-type cells [47,237,423]. Clearly, a fast resorption of brushite-forming formulations can only be achieved if the resorption occurs before conversion DCPD to CDHA according to Equation (14) [64]. Both types of the resorption mechanisms (active + passive) might occur almost simultaneously, if a hardened formulation consists of two different calcium orthophosphates, e.g., from DCPD and β-TCP. For example, the biphasic brushite-forming ChronOS^TM^ Inject was found to resorb by dissolution with cement disintegration and particle formation followed by the phagocytosis of the cement particles through macrophages [424]. Similar formulation was found to be degraded through a dissolution process associated with a cellular process. The observations suggested that cell activities could be influenced by a small particle size, without close correlation between the particle size and the cell activities but with a correlation between particle concentration and the cell activities [420]. To get further details on this topic, the interested readers are referred to an interesting review on the cellular degradation mechanisms of calcium orthophosphate bioceramics [425].

The summary of studies on brushite-forming formulations implantation in various animal models and defect locations is available in literature [259]. Generally, in the same animal model, a degradation rate decreases with a sample size increases, as does DCPD to CDHA conversion time. Data are available that hardened brushite-forming formulations experience an initial linear degradation rate of ~0.25 mm/week [266], which slightly overwhelms the bone regeneration capacity, resulting in small bone-material gaps and a reduction in mechanical properties [51]. The compositional changes of brushite-forming formulations after implantation in sheep is well described elsewhere [396,426].

The kinetics of passive resorption depends on porosity of the samples, ionic substitutions in calcium orthophosphates (when applicable), crystallinity and pH at the tissue interfaces. The active resorption is due to cellular activity; however, it is also related to the passive one. Namely, the solution pH near macrophages and osteoclasts can drop to ~5 by excretion of lactic acid, which increases the solubility (Figure 1), whereas near osteoblasts (bone forming cells) solution pH can become as high as 8.5 by excretion of ammonia [46]. Dissolution chemistry of CDHA (therefore, of hardened apatite-forming formulations) in acidic media [calcium orthophosphates are almost insoluble in alkaline solutions (Figure 1)] might be described as a slightly modified sequence of four successive chemical equations [427,428]:

Ca_10−*x*_(HPO_4_)*_x_*(PO_4_)_6−*x*_(OH)_2−*x*_ + (2–*x*)H^+^ = Ca_10−*x*_(HPO_4_)*_x_*(PO_4_)_6−*x*_(H_2_O)_2−*x*_^(2−*x*)+^ (15)

Ca_10−*x*_(HPO_4_)*_x_*(PO_4_)_6−*x*_(H_2_O)_2−*x*_^(2−*x*)+^ = 3Ca_3_(PO_4_)_2_ + (1 – *x*)Ca^2+^ + (2 – *x*)H_2_O (16)

Ca_3_(PO_4_)_2_ + 2H^+^ = Ca^2+^ + 2CaHPO_4_ (17)

CaHPO_4_ + H^+^ = Ca^2+^ + H_2_PO_4_^−^ (18)

Obviously, the dissolution chemistry of DCPD (therefore, of hardened brushite-forming formulations) in acidic media is described by Equation (18). One should stress, that in Equation (18) water is omitted for simplicity. Therefore, dissolution of DCPA is written instead.

Nevertheless, the situation with biodegradation mechanisms appears to be more difficult than it was expected before. Namely, in a special study brushite-forming MCPM/HA and MCPM/β-TCP formulations were compared to test the hypothesis that DCPD chemistry affected both degradation properties and cytocompatibility of the self-setting formulations [429]. Using simple *in vitro* models the authors found that brushite-forming MCPM/β-TCP formulations degraded primarily by DCPD dissolution, which was associated with a slight pH drop and relatively low mass loss. Cytocompatibility testing revealed no significant change in cell viability relative to the negative control for all of the MCPM/β-TCP formulations. In contrast, the brushite-forming MCPM/HA formulations were prone to undergo rapid conversion of DCPD to CDHA, resulting in a sharp pH drop and extensive mass loss. A stoichiometric excess of HA in initial formulations was found to accelerate the conversion process and significant cytotoxicity was observed. Presumably, the initial excess of HA promoted DCPD → CDHA transformation. The authors concluded that, although the product of the setting reaction was the same, brushite-forming formulations produced from MCPM/HA and MCPM/β-TCP differed significantly in their degradation properties and cytocompatibility [429].

**Figure 10:**
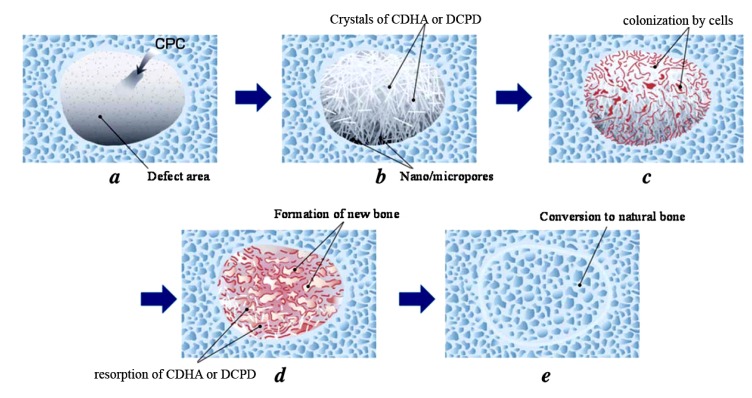
A schematic drawing of bone defect regeneration by means of a self-setting calcium orthophosphate formulation: (**a**) filling of a bone defect with a viscous formulation; (**b**) formulation setting with formation of a end product (CDHA or DCPD); (**c**) colonization by cells; (**d**) resorption of CDHA or DCPD by osteoclasts and bone formation by osteoblasts; (**e**) bone regeneration. Reprinted from [434] with permission.

The mechanism of bone healing caused by self-setting calcium orthophosphate formulations is very multifactorial because the surface of the formulations is rapidly colonized by cells. Several types of these cells degrade calcium orthophosphates by either phagocytotic mechanisms (fibroblasts, osteoblasts, monocytes/macrophages) or an acidic mechanism with a proton pump to reduce the pH of the microenvironment and resorb the hardened bioceramics (osteoclasts) [425,430]. Various mesenchymal cells located at the implantation sites can induce solubilization of calcium orthophosphates. Upon the cells arrival, various active enzymes, such as acid phosphatase, are secreted that causes dissolution of the hardened cements [431,432,433]. Much more biology, than chemistry and material science altogether, is involved into this very complex process and many specific details still remain unknown. Nevertheless, the entire process of bone defect healing by self-setting calcium orthophosphate formulation might be schematically represented by Figure 10 [434].

It is well known that various polypeptides and growth factors present in bone matrix might be adsorbed onto HA [435,436,437] and modulate the local milieu of cells. This is supported by many purification protocols of growth factors and bone morphogenetic proteins/osteogenins involving HA chromatography [438,439]. However, osteoblasts are not found in direct contact with calcium orthophosphates. A complex proteinaceous layer, usually osteoid, directly contacts the osteoblasts. After implantation of self-setting calcium orthophosphate formulations, mitogenic events could occur either during the initial mesenchyma1 cell contact or after osteoid degradation by osteoblast collagenase. In a dense, mineralized biomaterials such as hardened calcium orthophosphate formulations, which provide a barrier to the free diffusion of circulating hormones, growth factors, and cytokines, it is questionable whether the local responses at the periphery of the material regulate osteoconduction [22]. The tissue response to injectable calcium orthophosphate formulations is well described in literature [373,408,423,440,441]. Recent histological and mechanical evaluation in a sheep vertebral bone void model is available elsewhere [442]. The interested readers are also advised to get through a paper on the *in vitro* biodegradation of hardened brushite-forming formulations by a macrophage cell-line [152].

To conclude this part, one should note that self-setting calcium orthophosphate formulations are able to provide short-term biologically desirable properties and then be replaced by a new bone, which is very important [443]. In general, the growth rate of a newly forming bone depends on age, sex and general metabolic health of the recipient as well as on the anatomic site, porosity, bulk site, crystallinity, chemical composition (brushite or apatite), particle sizes and P/L ratio of the mixture. Considering all these factors, it might take from 3 to 36 months for different formulations to be completely resorbed and replaced by bones [228]. However, additional sound scientific data to determine the exact degree of biodegradability are still needed, viz. animal studies performed in a critical-size defect model. One must stress that the resorption kinetics should be balanced with the rate of new bone formation to avoid collapse at the fracture site, which might occur if the resorption is too fast. Interestingly that to advance self-setting calcium orthophosphate formulations as bioabsorbable bone replaceable materials, it is essential to utilize the patient’s own blood in combination with the formulations [444].

## 6. The Mechanical Properties

### 6.1. Nonporous Formulations

As in most clinical applications self-setting calcium orthophosphate formulations are applied in direct contact with human trabecular bones, it may be stated as a mechanical requirement that the strength of the formulations must be at least as high as that of trabecular bones, which is close to 10 MPa [445]. Due to a combination of different forces that may include bending, torsion, tension and compression, three-dimensional (3D) complex load is normally applied to human bones. Unfortunately, ordinary calcium orthophosphate cements are strong enough at compression only [223]. In theory, after setting, they can reach the mechanical properties comparable to those of calcium orthophosphate blocks with the same porosity. However, in practice, their strength is lower than that of bones, teeth or sintered calcium orthophosphate bioceramics [167].

**Figure 11:**
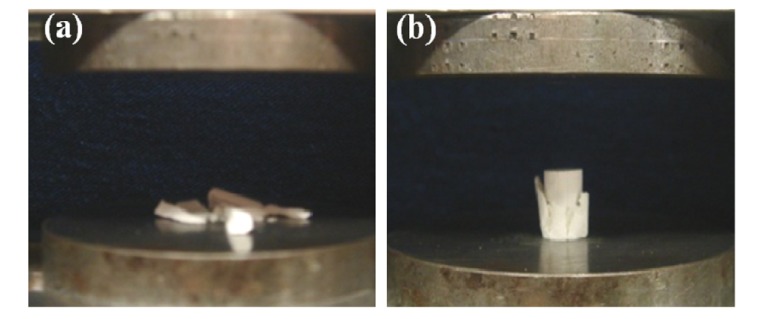
Pictorial representation of specimens post critical loading for (**a**) the control and (**b**) the same formulation reinforced by 5 wt% of bovine collagen fibers. Reprinted from [446] with permission.

Two types of mechanical assessments are usually performed with the hardened self-setting calcium orthophosphate formulations: compressive strength and tensile strength tests. Compressive strength measurements are performed on cylindrical samples with an aspect ratio of 2 until fracture occurs (Figure 11) [446]. On the other hand, direct tensile strength is difficult to measure in such brittle materials. Therefore, in many studies the alternative method of measuring the diametric tensile strength has been used, despite the fact that this technique gives results that underestimate the true tensile strength by a factor of 85% [447].

Having the ceramic origin, the set products of all calcium orthophosphate formulations are brittle, have both a low impact resistance and a low tensile strength (within 1–10 MPa), whereas the compression strength varies within 10 to 100 MPa [164,223,224]. The latter value exceeds the maximum compression strength of human trabecular bones. Furthermore, at 12 weeks after implantation the compressive strength of the hardened formulations was found to be still significantly higher (60–70 MPa) than that of normal bone [51]. In general, hardened brushite-forming formulations are slightly weaker than hardened apatite-forming ones. Namely, a tensile strength of ~10 MPa and a compressive strength of ~60 MPa were obtained for brushite-forming formulations [448]. In comparison, apatite-forming ones can reach a tensile strength of ~16 MPa [449] and a compressive strength of ~83 MPa [450]. However, due to the inherent brittleness of ceramics, those values are close to be meaningless. Namely, the indication of a mean compressive strength of, say, 50 MPa measured on well-prepared (e.g., under vibrations and pressure) and perfectly shaped samples does not inform the readers with which probability this formulation will fail *in situ* under a cyclic load of, e.g., 10 MPa. Furthermore, a comparison of the compressive strength of hardened formulations with that of cancellous bone is not very helpful either because cancellous bone is much less brittle than ceramics [149].

Moreover, the mechanical properties of hardened calcium orthophosphate formulations are not narrowly distributed around a mean value (as for metals), but widespread over a very large range of values, which strongly reduces their clinical application [451]. *In vivo*, the difference between the hardened apatite- and brushite-forming formulations boosts: namely, the mechanical properties of the former were found to increase [401], whereas those of the latter decreased [51]. This is attributed to a higher solubility of DCPD when compared with that of CDHA (Table 1). However, the mechanical properties of the hardened formulations may vary with implantation time. For example, animal studies indicated that the mechanical properties of apatite-forming formulations tended to increase continually [401], in contrast to those of brushite-forming ones, which initially decreased and again increased when bone was growing [51]. Furthermore, shear and tensile forces play a very important role. Thus, these parameters should also be considered, for example, using the Mohr circle approach [447]. Besides, it is difficult to compare the mechanical properties of different formulations. For example, the following numeric values of the compression strength and setting time were obtained: (i) Norian SRS^®^ (~50% porosity): 33 ± 5 MPa and 8.5 ± 0.5 min; (ii) Cementek^®^: 8 ± 2 MPa and 17 ± 1 min; (iii) Biocement D^®^ (~40% porosity): 83 ± 4 MPa and 6.5 ± 0.5 min; (iv) α-BSM^®^ (~80% porosity): 4 ± 1 MPa and 19 ± 1 min, respectively [450]. Among them, Biocement D^®^ has the highest compressive strength but the lowest porosity and a high compressive strength does not necessarily mean that Biocement D^®^ is the least breakable implant [4]. Additional details on the major properties of Norian SRS^®^ are available elsewhere [231,452]. Besides, the interested readers are suggested to get through the mechanical characterization of a bone defect model filled with ceramic cements [226].

To improve the mechanical properties of the self-setting calcium orthophosphate formulations, addition of water-soluble polymers might be considered. For example, in early 1990s, Miyazaki *et al*. [453,454] used a number of polymers, including polyacrylic acid and polyvinyl alcohol to improve the properties of a TTCP + DCPD formulation. They noted marked increases (up to threefold) in mechanical properties but with an unacceptable reduction of workability and setting time. Later, another research group reported similar results using sodium alginate and sodium polyacrylate [455]. Afterwards, other researchers added several polyelectrolytes, polyethylene oxide and a protein bovine serum albumin into α-BSM^®^ cement pastes to create calcium orthophosphate-polymer biocomposites [456]. Biocomposites of α-BSM^®^ with polycations (polyethylenimine and polyallylamine hydrochloride) exhibited compressive strengths up to six times greater than that of pure α-BSM^®^ material. Biocomposites of α-BSM^®^ with bovine serum albumin developed compressive strengths twice that of the original α-BSM^®^ [456]. Similar strengthening effect was achieved by addition of some commercial superplasticizers [457]. The results showed that small additions, *i.e*., 0.5 vol%, in the aqueous liquid phase improved the maximum compressive strength (35 MPa) of Biocement-H^©^ by 71%, *i.e*., till ~60 MPa. Moreover, the addition of high amounts of superplasticizers, *i.e*., 50 vol%, allowed for a significant increasing of the P/L ratio from 3.13 to 3.91 g/mL, without affecting the maximum strength and/or the workability [457]. This effect was explained by an inhibiting effect of the aforementioned additives on the crystal growth kinetics of newly forming crystals of calcium orthophosphates, which resulted in smaller crystallites and, hence, a denser and more interdigitated microstructure. However, the increased strength was attributed mainly to the polymer’s capacity to bridge between multiple crystallites (thus forming a more cohesive composite) and to absorb energy through a plastic flow [456]. Other factors affecting strength are the materials used in the solid phase, particle sizes, incorporation of fillers (see Section *7* for details), the P/L ratio and various additives to the liquid phase [130].

The strength of the cement-prosthesis interface might be studied by a pullout test. The details are available elsewhere [81].

### 6.2. Porous Formulations

As presence of pores simplifies for cracks to run throughout the ceramic mass, the mechanical properties of the hardened formulations were found to decrease exponentially with the porosity increase [458]. In theory, self-setting calcium orthophosphate formulations can be made with almost any porosity. However, for most commercial formulations, the pores are of 8–12 μm in diameter and, after setting, porosity occupies about 40%–50% of the entire volume [459]. To reduce the porosity of hardened formulations, pressure can be applied [167,460,461]. Usually, the pore dimensions in hardened formulations are too small to allow a fast bone ingrowth. Thus, there is a lack of macroporosity. Besides, unless special efforts have been performed, the available pores are not interconnected. Due to these reasons, after injection, osteoclastic cells are able to degrade the hardened calcium orthophosphates layer-by-layer only, starting at the bone/implant interface throughout its inner part (in other words, from the outside to the inside). This is the main drawback of the classical self-setting formulations when compared to calcium orthophosphate ceramic scaffolds with an open macroporosity [4,5].

**Figure 12:**
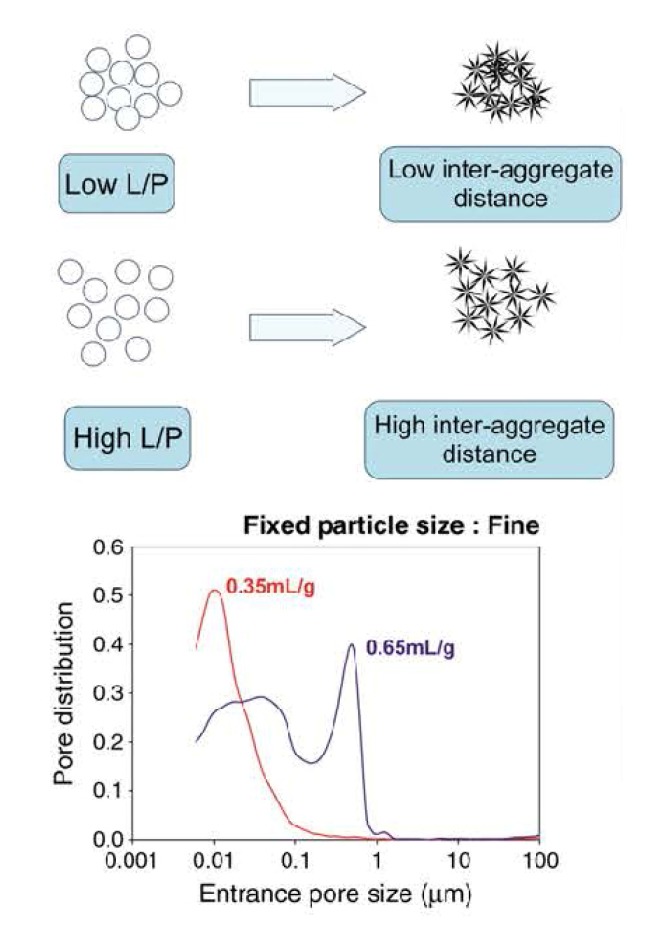
A schematic drawing of the influence of the L/P ratio on the properties of self-setting formulations. Reprinted from [219] with permission.

Since strength is reciprocally proportional to porosity [417], the former might be adjusted by varying the P/L ratio in the hardening mixtures. At high P/L (*i.e*., low L/P) ratios the space between particles in self-setting pastes decreases. Considering that precipitation of new crystals takes place surrounding the initial powder particles, this leads to a more compact structure of the crystal agglomerates (Figure 12) [219]. Elevated compression strength would be applicable in cranioplasty for regions requiring significant soft-tissue support. For small bone defects, such as root canal fillings, formulations of low compression strength might be used [160]. Concerning the tensile strength of self-setting calcium orthophosphate formulations, as a rule of thumb, it appears to increase two-fold with each 10 vol% decrease of the porosity, *i.e*., 5, 10, 20, 40 and 80 MPa for 80%, 70%, 60%, 50% and 40% porosity, respectively [4,5]. The effect of porosity on the compressive modulus of self-setting calcium orthophosphate formulations is available as Figure 4 in [461]. Ishikawa and Asaoka showed a linear relation (*R*^2^ = 0.94) between the natural logarithm of diametral tensile strength and porosity of self-setting calcium orthophosphate formulations where porosity was controlled by compaction pressure (up to 173 MPa) [164]. Besides, an empirical relationship between strength, *S*, and porosity, *P*, has been introduced in another study:

*S* = *S*_0_·e^−*bP*^

where *S*_0_ is the theoretical strength at *P* = 0 (fully dense) and *b* is an empirical constant [462].

As the porosity is mainly due to an excess of water used in the self-setting formulations, attempts were made to reduce the amount of water. However, the amount of water determines the rheological properties of self-setting pastes: a decrease in water content leads to a large increase in viscosity, eventually leading to non-flowable pastes. As calcium orthophosphate formulations are set at an almost constant volume, the final porosity can be predicted from the initial composition [4,5]. A shrinkage degree of ~1% causes no restrictions on clinical use [221]. Studies on the *in vivo* evaluation of an injectable macroporous calcium orthophosphate formulations revealed a higher bioresorption rate due to both a higher surface contact with body fluids (which increases dissolution) and enhancing cellular activity due to particle degradation [311,373].

Besides the addition of porogens [337,338,339,340,341,342,343,344,345,346,347,348,349,350,351,352,353,354,355,356,357,358,359,360,361], the porosity level of the self-setting calcium orthophosphate formulations might be controlled to a certain extent by adjusting particle sizes and the P/L ratio. When the P/L ratio is high, the porosity of the hardened formulations is low [4,5]. According to calculations, the tensile strength of the formulations with zero porosity could be as high as 103 MPa [164]. However, a high density and a lack of pores decreases bioresorbability because a newly forming bone appears to be unable to grow into the implant; it might grow only simultaneously with dissolution of the hardened formulations. Thus, porosity of self-setting calcium orthophosphate formulations is a very important factor for their biodegradability [4,5].

## 7. Reinforced Formulations and Concretes

Being aware on the excellent bioresorbability of DCPD and CDHA, researchers are focused on attempts to overcome the mechanical weakness of the self-setting calcium orthophosphate formulations by using different fillers, fibers and reinforcing additives that give rise to formation of various multiphasic biocomposites [128,129,133,225,296,459,462,463,464,465,466,467,468,469,470,471]. Even carbon nanotubes have been successfully tested to reinforce the self-setting formulations [29,472,473,474]. Although the biomaterials community does not use this term (just 1 paper has been published [475]), a substantial amount of such formulations might be defined as calcium orthophosphate concretes. According to Wikipedia, the free encyclopedia: “Concrete is a construction material that consists of a cement (commonly Portland cement), aggregates (generally gravel and sand) and water. It solidifies and hardens after mixing and placement due to a chemical process known as hydration. The water reacts with the cement, which bonds the other components together, eventually creating a stone-like material” [476]. The idea behind the concretes is simple: if a strong filler is present in the matrix, it might stop crack propagation. In such formulations, the load is transferred through the matrix to the fillers by shear deformation at the matrix/filler interfaces. Both fillers and matrix are assumed to work altogether providing a synergism needed to make an effective composite. However, adding fillers always reduced porosity, which negatively influenced the ability of the concretes to allow bone ingrowth into the pores. Hence, denser formulations have slower resorption rates and thus a slower bone substitution [164]. Moreover, due to the presence of fillers, injectability and other rheological properties of the reinforced formulations and concretes frequently appear to be worse than the same properties of the ordinary formulations. Thus, it is difficult to increase strength of the self-setting formulations without having a negative influence on the other properties.

The reinforced formulations and concretes can be prepared from both apatite-forming and brushite-forming formulations. For example, in an attempt to improve the mechanical properties of calcium orthophosphate formulations, a group of investigators prepared concretes by adding human cadaveric femur bone chips in amounts of 25%, 50% and 75% (w/w) to α-BSM^®^ cement [464]. The mechanical tests revealed that the specimens of pure cement exhibited a relatively high stiffness but a low ductility. However, for the concretes an increasing of bone content was found to result in the elastic modulus decreasing and the ductility increasing; however, the ultimate strength showed only small changes with no apparent trend [464]. A concrete of Biopex^®^ cement with allografts taken from femurs and tibiae of rabbits is also available. Unfortunately, nothing is written on the mechanical properties improvement but, surprisingly, by the addition of allografts, the hydrolysis process of Biopex^®^ was significantly changed [296]. By adding polymers, other researchers succeeded in improving the mechanical strength of the formulations up to ~30 MPa; however, both the kinetics of CDHA formation and, thus, the bioactivity were decreased [134,477]. Xu *et al*. [478] reported that incorporation of long carbon fibers at a volume fraction of 5.7% increased the flexural strength about 4 times and work of fracture ~100 times, if compared to un-reinforced formulations. In another study, DCPD-forming formulations were reinforced by poly(propylene fumarate) and, if compared with non-reinforced controls, flexural strength improved from 1.80 ± 0.19 to 16.1 ± 1.7 MPa, flexural modulus increased from 1073 ± 158 to 1304 ± 110 MPa, maximum displacement during testing increased from 0.11 ± 0.04 to 0.51 ± 0.09 mm and work of fracture improved from 2.7 ± 0.8 to 249 ± 82 J/m^2^ [479]. The reinforcement mechanisms were found to be crack bridging and fiber pullout, while fiber length and volume fraction were key microstructural parameters that determined the concrete properties [480]. Although addition of polypropylene, nylon and carbon fibers was found to reduce the compression strength of a double-setting calcium orthophosphate formulation due to increased porosity, it strongly increased the fracture toughness and tensile strength, relative to the values for the un-reinforced formulations [465]. A knitted two-dimensionally oriented polyglactin fiber-mesh was found to be effective in improving load-bearing behavior of self-setting formulations for potential structural repair of bone defects [225]. To make the material stronger, fast setting and anti-washout, chitosan might be added [202,389,453,480,481,482,483,484,485,486,487,488,489,490]. Furthermore, anti-washout properties might appear by adding sodium alginate [491]. Calcium orthophosphate concretes containing SiO_2_ and TiO_2_ particles showed a significant (~80–100 MPa) increase in the compressive strength, whilst no change in the mechanical behavior was observed when ZrO_2_ particles were added [492]. Additional examples of the properties improving comprise addition of calcium silicates [83], calcium carbonate [71,492], polypeptide copolymers [493], fibrin glue [494] and collagen [446,495,496,497,498,499,500,501,502,503]. Figure 11 shows specimens of an hardened unreinforced calcium orthophosphate formulation (a) and the same formulation reinforced by 5 wt% of bovine collagen fibers (b) after compression loading up to ~15% level of strain. The characteristic brittle behavior of the set unreinforced formulation can be observed, as the specimen exhibited catastrophic failure after critical loading and subsequently broke into fragments. Observing a typical compression specimen of the set reinforced formulation after testing clearly displays that the failure mechanism was very different, as the specimen maintains a degree of cohesive structure and remains capable of supporting a load [446]. Additionally, strength improvement was found when DCPA and TiO_2_ crystals were used as fillers for mechanically activated α-TCP formulations [504].

**Figure 13:**
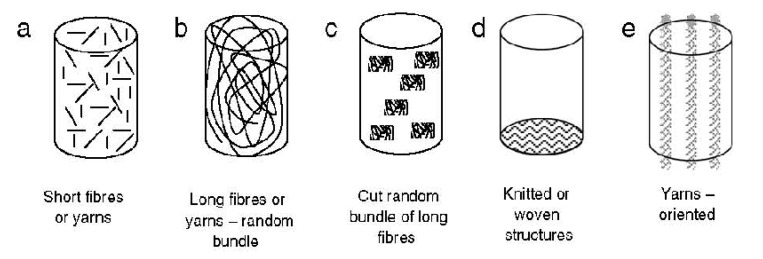
Different ways of fiber disposition in the fiber-reinforced calcium orthophosphate self-setting formulations. As seen from this figure, fibers can be introduced as short staple ones (**a**) or as long fibers forming a random bundle (**b**). The random bundles can also be cut into small pieces and dispersed inside the self-setting matrix (**c**). If fibers are spun into yarns, the latter can also be cut and introduced randomly into the formulation [(**a**), (**b**)], oriented (**e**) or may be woven or knitted into laminar textile structures (**d**). Reprinted from [505] with permission.

The blending of fibers with the self-setting pastes or precursor powders can be carried out using different structures of the fibrous materials, as shown in Figure 13 [505] and the effects of varying fiber type, fiber length and volume fraction of fiber-reinforced calcium orthophosphate formulations were investigated [481,506]. Four fiber types were studied: aramid, carbon, E-glass and polyglactin. Fiber length ranged within 3–200 mm and fiber volume fraction ranged within 1.9%–9.5%. The results indicated that self-setting formulations were substantially strengthened via fiber reinforcement. Aramid contributed to the largest increase in strength, followed by carbon, E-glass and polyglactin. Fiber length, fiber volume fraction and fiber strength were found to be key microstructural parameters that controlled the mechanical properties of the concretes [481,506]. Fiber reinforcement of porous formulations (mannitol was used as a porogen) was performed as well [507]. Namely, reinforcement by aramid fibers (volume fraction of 6%) was found to improve the properties of a calcium orthophosphate concrete with the strength increasing threefold at 0% mannitol, sevenfold at 30% mannitol and nearly fourfold at 40% mannitol. Simultaneously, the work of fracture increased by nearly 200 times, however the modulus was not changed as a result of fiber reinforcement [507]. Addition of 20 wt% of acrylamide and 1 wt% ammonium polyacrylate to the liquid increased the compressive and tensile strength of α-TCP formulation by 149% and 69% (55 and 21 MPa), respectively due to a dual setting mechanism (*i.e*., both inorganic and organic phases were set simultaneously) [508]. Other types of the dual setting formulations containing calcium orthophosphates are known as well [509,510,511]. A positive influence of polyamide fibers [512] and bioactive glass [513,514,515,516] is also known. Interestingly that reinforcement of self-setting formulations might be performed by infiltration of a preset composition by a reactive polymer and then cross-linking the polymer *in situ* [517].

The reinforcement of self-setting calcium orthophosphate formulations by resorbable or biodegradable fillers responds to a different strategy. In this case, the rationale is to provide strength augmentation at the initial stages and, subsequently to filler degradation, to facilitate bone ingrowth into the macropores [362,363,364,365,366,367,368,369,370]. For example, the initial strength of a concrete was threefold higher than that of the unreinforced control [362]. The work of fracture (toughness) was found to increase by two orders of magnitude for other biocomposites of calcium orthophosphates with resorbable fibers, such as Vicryl (polyglactin 910) (Ethicon, Somerville, NJ, USA) [363] and a mesh of copolymer of polyglycolic and polylactic acids [367]. The addition of fillers with higher bioresorption rate than the hardened calcium orthophosphate matrix allows creating macropores to favor cell colonization, angiogenesis and eventually fostering bone regeneration. Ideally, the loss of strength produced by filler degradation should be compensated by the formation of new bone. One important advantage of long fibers over particulates and short fibers is the fact that once resorbed they form a network of interconnected channels inside the set structures, which could facilitate bone ingrowth into implants [166,167,362,367]. For example, interconnected macropores were formed in a hardened formulation at 84 days’ immersion in a physiological solution [367]. One should note that, apart from the mechanical properties of the reinforcing materials, the structure of the incorporated fibers, regular or random, appears to be crucial for the resulting flexural strength and modulus of elasticity [365]. A higher strength might help extending the use of calcium orthophosphate formulations to larger stress-bearing repairs, while the macropores might facilitate tissue ingrowth and integration of the hardened formulations with adjacent bones. To extend this idea further, several types of fibers with different rates of bioresorbability might be simultaneously incorporated into self-setting formulations.

Besides the aforementioned, it is important to mention on the reinforced formulations and concretes, after hardening consisting of calcium orthophosphates only [266,267,424,518,519,520,521,522,523]. The first biphasic concrete consisting of a hardened DCPD matrix filled with β-TCP granules was introduced in 1992 [519]. Further development of this formulation was well described in other papers [266,424]; unfortunately, neither mechanical nor rheological properties of that concrete were disclosed. Nevertheless, the results of still another study showed that, by addition of 20 wt% the as-prepared β-TCP aggregates, the compressive strength of the self-setting concrete was increased by about 70%, while the paste itself still maintained injectable, while the heat release in the hydration process decreased by ~25% [522].

At physiological pH, the *in vitro* solubility of DCPD is approximately 100 times higher than that of β-TCP (Table 1 and Figure 1); roughly, the same order of magnitude applies for the *in vivo* resorption kinetics of these calcium orthophosphates. Thus, a new bone is formed in the space left after resorption of the DCPD matrix, while β-TCP granules act as guiding structures. This feature of the cement can be considered an inverse scaffolding effect [524]. Another group of investigators invented a formulation that incorporated as major powder components α-TCP, ACP and BCP (HA + β-TCP in various HA/β-TCP ratios) [463]. It was believed that after setting such a formulation could provide a porous bioceramics *in vivo* due to preferential dissolution of a better soluble ACP component compared to the other calcium orthophosphates in the matrix. Further, this combination was extended to a multiphase concrete composition consisting of 70% w/w settable matrix (mixture of 45% α-TCP, 5% MCPM and 25% of ion-substituted ACP) with the average particle dimensions of 15 µm and 30% BCP (HA + β-TCP) granules (ranging between 80 and 200 µm) as a filler [518]. The role of BCP granules is quite interesting: after implantation of a formulation without BCP granules, the quality of newly formed bone was not identical to the host bone, while implantation of a concrete with BCP granules resulted in formation of a new bone identical to the host bone. The reason of this phenomenon is not clear yet; but, perhaps, it correlates with similar results for β-TCP granules, which act as bone anchors and encourage formation of a mature bone [266,267]. Other ACP-containing formulations were elaborated as well [525].

Effects of added α-TCP and β-TCP were investigated to shed light on the setting reactions of apatite-forming formulations consisting of TTCP and DCPA [521]. Added β-TCP showed no reactivity, and thus resulted in extended setting time and decreased mechanical strength. In contrast, α-TCP dissolved to supply calcium and orthophosphate ions after initial apatite crystal formation by the chemical reaction (1). Although setting time was delayed because α-TCP was involved only in the latter reaction of apatite cement, larger apatite crystals were formed due to its addition. Due to larger apatite crystal formation, the mechanical strength of the α-TCP-added formulations increased by approximately 30%, as compared to α-TCP-free ones [521]. In another study, HA whiskers were used as the reinforcement phase to prepare concretes and the maximum strength was achieved when HA whiskers were added in amount of 4 wt% [523]. Besides, self-setting calcium orthophosphate formulations might be reinforced by calcium polyphosphate fibers [526,527]. Additional details on this topic might be found in special reviews [505,528].

To conclude this part, one should briefly mention on the reverse situation: there are bone concretes made of various polymeric cements, reinforced by calcium orthophosphate powders or granules to establish a compromise between the desired mechanical and biological properties [529,530,531,532,533,534,535,536]. The calcium orthophosphates presented in such formulations act as fillers, which are necessary to both improve the mechanical properties and impart bioactivity; however, they do not participate in the hardening mechanisms. For example, the higher the amount of HA was in bioactive acrylic bone cements, the higher were the compressive and tensile moduli. Furthermore, as the percentage of HA increased to 20 wt%, the heterogeneity of the material was higher [534]. Polymerization of monomers is primarily responsible for setting of such types of biocomposites and concretes. However, that is another story.

## 8. Biomedical and Clinical Applications

Injectable and self-setting calcium orthophosphate formulations have been introduced as adjuncts to internal fixation for treating selected fractures. Different studies have already shown that they are highly biocompatible and osteoconductive materials, which can stimulate tissue regeneration [22,537]. The main purpose of calcium orthophosphate cements, concretes, pastes and putties is to fill voids in metaphyseal bone, thereby reducing the need for bone graft, although such formulations might also improve the holding strength around metal devices in osteoporotic bone. Bone augmentation (*i.e*., a reinforcement of osteoporotic bone through injection) appears to be a very promising application field of the self-setting calcium orthophosphate formulations. Such procedures ease the fixation of screws in mechanically poor bone (for example for osteosynthesis) and decrease pains associated with unstable vertebrae. The combination of a self-setting nature, biocompatibility, lack of any by-products and a great potential for replacement by bones make calcium orthophosphate cements, concretes, pastes and putties very promising materials for clinical and medical applications. In addition, they can easily be used by bone remodeling cells for reconstruction of damaged parts of bones [126,127,255,441,538,539,540]. The ability to be molded in place also is a very important property because these formulations can easily be delivered into the desired place and can be fitted perfectly with bone defects [127]. Besides, some formulations were found to possess an antimicrobial activity [72,75,77,84,541], as well as promote osteoblast cell adhesion and gene expression *in vitro* [542].

Numerous studies reported optimistic results on the clinical application of the self-setting calcium orthophosphate formulations. For example, the data on cytocompatibility and early osteogenic characteristics are available in literature [543]. The ratio of the cases determined to be “effective” or “better” among the 74 cases we found to be 97.3% [544]. Besides, the results of intra-articular degradation and resorption kinetics of these formulations revealed no signs of pronounced acute or chronic inflammation [545]. Injected Norian SRS^®^ cement was mainly found as a single particle, anterior to the cruciate ligaments. Synovial tissues surrounded the cement within 4 weeks and signs of superficial resorption were found [545]. However, disintegration or washout of self-setting calcium orthophosphate formulations has been reported as a potential clinical problem [164,238]. Perhaps, this problem could be solved by putting pressure on the paste during the setting period. In addition, sodium alginate might be added; however, the mechanical properties (strength) of this formulation are still poor [132].

According to the available information, the earliest attempts for biomedical applications of the self-setting calcium orthophosphate formulations occurred in 1984 and were related to dentistry [546,547]. However, those were *in vitro* studies, while the earliest animal studies were performed in 1987 [32]. Afterwards, in 1991, a TTCP + DCPA cement was investigated histologically by implanting disks within the heads of nine cats [548]. Simultaneously, another research group evaluated the tissue reactions to this cement in the teeth of monkeys [549]. Some important examples of medical applications of the self-setting calcium orthophosphate formulations are given below.

### 8.1. Dental Applications

A group of investigators extracted all mandibular premolar teeth from beagles [550]. After one month of healing, alveolar bone was reduced to make space for a previously fabricated calcium orthophosphate cement block. One more month later, 8-mm HA implants were placed in such a manner that the apical half was embedded into alveolar bone and the coronal half in the calcium orthophosphate cement block. The investigators observed that the cement block was gradually replaced by bone and histopathologic features of the cement area were similar to that of natural bone. Moreover, the coronal half of the implants, previously surrounded by the calcium orthophosphate cement, was firmly attached by natural bone [550]. In another study, the same researchers used fluorescent labeling analysis and electron microanalysis to measure the extent of new bone formation and elemental (Ca, P, Mg) distribution [551]. The results indicated the presence of newly formed bone at ~1 month after surgery and similar elemental distributions in the calcium orthophosphate cement and natural bone areas at ~6 months after surgery [228].

A self-setting calcium orthophosphate was injected as a bone filler for gaps around oral implants placed on the medial femoral condyles of six goats and found excellent bone formation around the graft material. Unfortunately, the degradation rate of the formulation appeared to be very slow and no resorption was observed [552]. In another study, a self-setting formulation was placed on artificially created periodontal defects but no significant difference was found between the hardened formulation and control. However, the formulation acted as a scaffold for bone formation and provided histocompatible healing of periodontal tissues [553]. Still other investigators used a self-setting formulation for direct pulp capping [554,555] and compared it to calcium hydroxide. Both materials were found to be equally capable of producing a secondary dentin at ~24 weeks [555]. Positive results were obtained in other studies [156,556]. Besides, self-setting calcium orthophosphate formulations were tried as root canal fillers [75,557,558], for pulpotomy [559] and restoration of enamel carious cavities [37]. Finally, self-setting calcium orthophosphate formulations can be used as adjunctive supportive agents for dental implants [560]. Further details on the dental applications of calcium orthophosphates might be found in a topical review [561].

### 8.2. Oral, Maxillofacial and Craniofacial Applications

Bone regeneration in oral, maxillofacial and craniofacial surgery can be divided in two main types of procedures: bone augmentation and bone defect healing. The use of self-setting calcium orthophosphate formulations for such purposes seems logical, as there is little or no stress generated under these conditions. Moreover, the ability to mold the material at placement is an enormous advantage from a cosmetics standpoint [228]. For example, BoneSource^TM^ is indicated for the repair of neurosurgical burr holes, contiguous craniotomy cuts and other cranial defects with a surface area no larger than 25 cm^2^ per a defect. In addition, it may be used in the sinus region for facial augmentation [127,562] and the formulation can be supported by metal hardware [127]. In dogs, BoneSource^TM^ was employed to supplement the supraorbital ridge and to augment skull base defects [563]. Another group performed trials to ascertain the inflammation around the site and the degree of loss of the implanted BoneSource^TM^. The material was found to be osteoconductive with both periosteal and endosteal bone formation [564]. Still another group presented excellent results using the material combined with an underlying resorbable mesh in calvarian defects of Yorkshire pigs. They found progressive bone ingrowths in all defects at 180 days, with nearly complete replacement by host bone [368]. Besides, excellent results for over 100 human patients were reported when a self-setting calcium orthophosphate formulation was used in cranial defects. The success rate after 6 years was 97% [118]. Furthermore, self-setting calcium orthophosphate formulations are used in orbital reconstructions [141,143,565]. The results of still other medical trials are available elsewhere [248,566,567,568,569,570,571,572,573,574,575,576,577,578,579,580].

To conclude this part, one should stress that complications still occur: namely, two cases of apatite-forming cement resorption and subsequent seroma formation have been reported for patients who had undergone retrosigmoid craniotomy [581]. Furthermore, another study describes complications occurred with 17 patients who underwent secondary forehead cranioplasty with Norian^®^ CRS [582]. Of 17 patients, 10 (59%) ultimately had infectious complications. Infection occurred on a mean of 17.3 months after surgery and of the 10 patients with complications, 9 required surgical debridement and subsequent delayed reconstruction. The authors concluded that although apatite-forming cements could yield excellent aesthetic results, their use in secondary reconstruction yielded unacceptably high infection rates leading to discontinuation of their use in this patient population [582].

### 8.3. Orthopedic Applications

Self-setting calcium orthophosphate formulations have successfully been used for treatment of the distal radius fracture [234,583,584]. Besides, other successful attempts have been made to use these formulations for calcaneal fractures [585], hip fractures [586,587], augmentation of osteoporotic vertebral bodies [588], distal radius fractures [589], tibial plateau fractures [50,589,590,591,592,593,594], restoration of pedicle screw fixation [595,596], reinforcement of thoracolumbar burst fractures [597], cancellous bone screws [598,599], vertebral body fillings [600], in wrist arthrodesis [601] and for fixation of titanium implants [602]. A study on a cement augmentation of the femoral neck defect might be found elsewhere [603]. Considering their properties, self-setting calcium orthophosphate formulations might potentially be applied to reinforce osteoporotic vertebral bodies [588,604]. Further details and additional examples on this topic are available elsewhere [248,605,606,607]. Besides, the self-setting formulations appear to be a reliable subchondral replacement biomaterial when the bone defect is adjacent to the articular cartilage [608].

### 8.4. Vertebroplasty and Kyphoplasty

Vertebroplasty and kyphoplasty are two surgical procedures that recently have been introduced to medically manage of osteoporosis-induced vertebral compression fractures. Particularly, both procedures aim to augment the weakened vertebral body, stabilize it and/or restore it to as much of its normal height and functional state as possible. Both procedures involve injection of self-setting calcium orthophosphate pastes into the fractured vertebral body, which resulted in a faster healing [118,231,608,609,610,611,612,613,614,615,616]. Furthermore, prophylactic injections of such formulations also have been performed.

### 8.5. Drug Delivery

In general, a potential substrate to be used as a drug carrier must have the ability to incorporate a drug, retain it in a specific target site and deliver it progressively with time in the surrounding tissues. Therefore, a certain level of porosity is mandatory. Additional advantages are provided if the biomaterial is injectable, biodegradable, sets at ambient temperature, has both near neutral pHs and a large surface area [52,53]. These properties make self-setting calcium orthophosphate formulations to be very attractive candidates as drug carriers for therapeutic peptides [617], antibiotics [26,618,619,620,621,622,623,624,625,626,627,628,629], anticancer [630,631,632,633,634] and anti-inflammatory agents [635,636], cytokines [637], hormones [638], bone morphogenetic proteins [488,639,640,641,642,643] and other biologically active compounds [644,645,646,647,648,649,650,651]. For example, a “growth factor cement” has been reported [652]. In that study, a combination of bone morphogenetic protein-2 (BMP-2), transforming growth factor-beta (TGF-β1), platelet-derived growth factor and basic fibroblast growth factor (bFGF) was used in a calcium orthophosphate cement for treatment of peri-implant defects in a dog model. The findings indicated a significant effect of the “growth factor cement” on increased bone-to-implant contact and amount of bone per surface area if compared with both the cement-only and no-cement treatment groups [652]. Similar data were found for a combination of a self-setting formulation with an exogenous nerve growth factor [653]. Even more complicated combination of deproteinized osteoarticular allografts integrated with a calcium orthophosphate cement and recombinant human vascular endothelial cell growth factor plus recombinant human BMP-2 (rhBMP-2) has been studied as well [654]. The drug delivery properties of the self-setting calcium orthophosphate formulations might be influenced by crystal morphology, porosity and microstructure [655].

**Figure 14:**
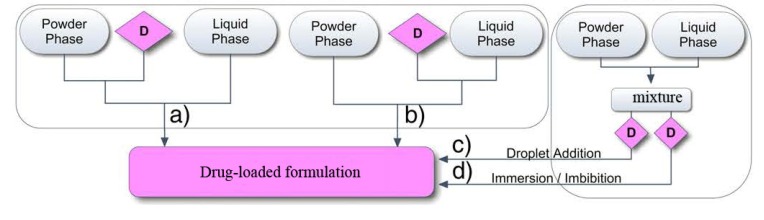
A schematic drawing of the possible ways of drug (denoted as D) incorporation into the self-setting formulations. Prior phase mixing a drug can either be distributed within the powder phase (**a**) or solubilized in the liquid phase (**b**). Drug loading can also be made after setting by droplet addition (**c**) or by imbibition (immersion) in the drug-containing solution (**d**). The procedures (c) and (d) do not allow injection since they require formulation pre-setting. Reprinted from [219] with permission.

**Figure 15:**
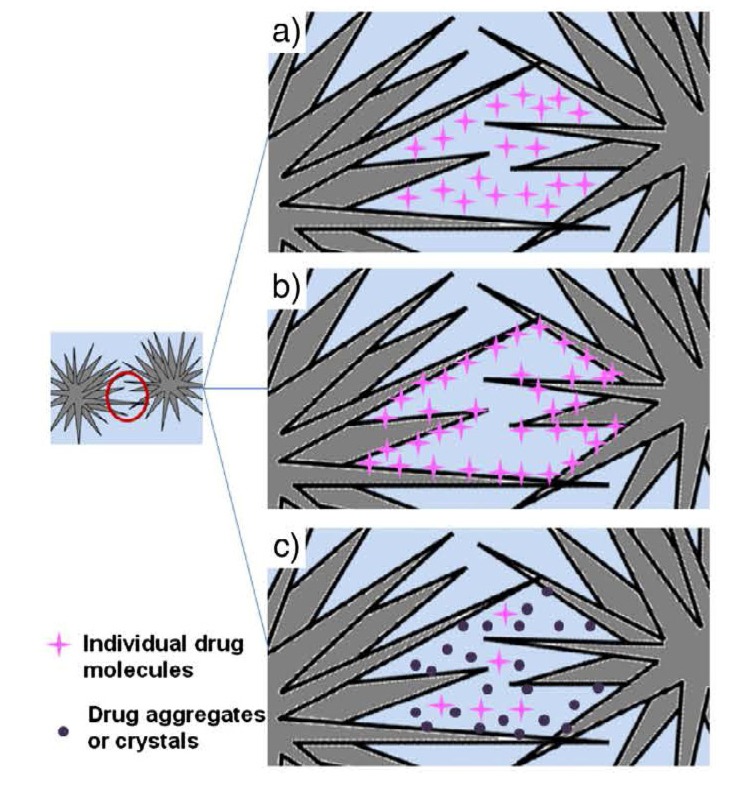
A schematic drawing of the different ways a drug can be found within a solid matrix: (**a**) as individual molecules dissolved in the remaining liquid within the pores; (**b**) adsorbed or chemically bound to the crystals surface; (**c**) in a solid form, as drug crystals or aggregates. Reprinted from [219] with permission.

For the self-setting formulations, the first issue that has to be considered and which will determine a drug distribution and its interaction with the matrix is the incorporation method of the drug. In principle, drugs (as well as hormones, cells and other biomedical or biological compounds) might be incorporated into both a liquid and a powder phases before phase mixing, as well as into the self-setting formulations obtained after both phases have been mixed. This process is schematically shown in Figure 14 [219]. After setting, the drugs appear to be distributed within a porous solid matrix. According to a topical review on the subject [219], there are 3 options of drug existence inside the matrix: (a) dissolved in the remaining liquid phase within the existing pores among the newly formed inorganic crystals; (b) adsorbed or chemically bound on the surface of the crystals; or (c) in a solid form inside pores (Figure 15).

**Figure 16:**
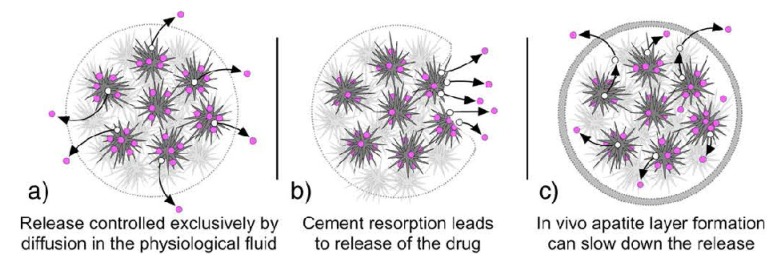
A schematic drawing of the different ways a drug release from hardened formulations. Reprinted from [219] with permission.

Studies on drug release are the second most important topic on drugs incorporation into the self-setting formulations [238,623,624,625,626,656,657]. This process is regulated by the microstructure of the set formulations (*i.e*., their porosity), as well as by presence or absence of additives able to influence the movement of drug molecules within the solid matrix. For example, it was observed that hardened formulations with very low porosity showed much slower drug release patterns than those with higher porosities [624]. Moreover, drugs that inhibit the setting reactions and reduce the porosity have a slower rate of release. This phenomenon has been observed with gentamicin sulfate. The presence of sulfate ions in this drug inhibits brushite crystal growth, resulting in a finer solid microstructure with lower porosity that slows down drug release [619]. In another study, a group of investigators added flomoxef sodium to a self-setting formulation and found that the release of antibiotic could be easily controlled *in vivo* by adjusting the content of sodium alginate [238]. *In vitro* elution of vancomycin from a hardened cement has been studied as well [657]. Concerning possible mechanisms of the drug releases, a topical review on the subject [219] describes three reasonable scenarios: (a) if the rate of the matrix degradation is slower than drug diffusion, drug release is controlled by diffusion of the drug through the liquid permeating the set formulation (might be valid for apatite-forming formulations); (b) if the rate of matrix degradation is faster than drug diffusion, the former controls drug release (might be valid for brushite-forming formulations) and (c) in some cases, an apatite layer can be formed on the surface after implantation, this hindering the diffusion of the drug to the surrounding tissue (Figure 16). Finally, various types of surface coatings might be applied as well to slow down the kinetics of drug release.

Studies on drug adsorption appear to be the third most important topic. In general, adsorption of any type of bioorganic molecules is related to chemical interactions between their functional groups and the calcium orthophosphate matrix; the strength of this interaction will influence the release pattern of drugs. Accordingly, bioorganic substances with adherent functional groups show a slow release pattern, whereas those that do not adhere well to calcium orthophosphate matrices will be more rapidly released. For instance, vancomycin release from 3D printed brushite matrices is complete within 1–2 days, while only 25% of tetracycline loaded on the same matrix is released after 5 days incubation [658]. Therefore, in order to obtain adequate release patterns, adsorption of bioorganic molecules to the self-setting matrix needs to be tuned. This can be done by either selecting the most appropriate drug for the matrix or modifying the self-setting matrix itself. For instance, doping brushite-forming formulations with Sr was found to reduce the antibiotic adsorption capacity, resulting in an increase in the fraction of drug released and in a faster release rate [659].The laboratory studies on drugs incorporation into the self-setting calcium orthophosphate formulations cover different aspects. Firstly, it is necessary to verify that addition of a drug does not influence the setting reaction not only in terms of the setting and hardening mechanisms but also with respect to the rheological behavior and injectability; Secondly, it is necessary to determine the *in vitro* kinetics of drug release; Thirdly, the drug delivery properties of the formulation must be studied *in vivo*. Finally, but still importantly, the clinical performance of the drug delivery system must be evaluated as well [52,53]. For example, recombinant human transforming growth factor β1 (rhTGF-β1) was added to a calcium orthophosphate cement [660,661,662,663]. This resulted in formation of a bioactivated formulation that could be used as a bone filler and for the replacement of bone [660]. It appeared that after 8 weeks the addition of growth factors stimulated and increased bone formation (50% volume) and bone contact (65%) in comparison to control calvarian defects in an animal study. Besides, the growth factor group reduced the remaining volume of the cement by 20% [661]. Examples of rhBMP-2 release from a loaded porous calcium orthophosphate cement might be found elsewhere [663,664], while an experimental study on a self-setting formulation impregnated with dideoxy-kanamycin B is also available [665]. In addition, self-setting calcium orthophosphate formulations might be loaded by other bioactive and/or biological compounds, such as nucleic acids [666]. Further details and additional examples are well described elsewhere [46,52,53,219]. Thus, the possibility of using injectable and self-setting calcium orthophosphate formulations as drug-delivery systems offers an attractive and efficient solution for the treatment of various bone diseases, e.g., tumours, osteoporosis and osteomyelitis, which normally require long and painful therapies.

### 8.6. Brief Conclusions on the Biomedical Applications

To conclude the biomedical part, one should stress that despite several encouraging results, not every surgeon’ expectation has been met yet [665]. First, self-setting calcium orthophosphate formulations are not superior to autografts, despite offering primary stability against compressive loading [667,668]. One of the main concerns of clinicians is to reach higher rates of bioresorption, an improvement of bone reconstruction and to a lesser extent, higher mechanical resistance [50]. Besides, clinical application of the self-setting formulations in comminuted fractures revealed penetration of the viscous paste into the joint space [669,670,671]. The interested readers are referred to a paper on cement leakage during vertebroplasty [672]. To date, cadaveric studies have already shown that using of the self-setting formulations with conventional metal fixation in certain fractures of the distal radius, tibial plateau, proximal femur and calcaneus can produce better stability, stiffness and strength than metal fixation alone. Early clinical results have revealed a reduced time to full load bearing when the formulations were used for augmentation of tibial plateau and calcaneal fractures, more rapid gain of strength and range of motion when used in distal radius fractures and improved stability in certain hip fractures [539,583]. However, surgeons reported on difficulties in filling the vertebral bodies (a bad injectability of present formulations) and other problems, such as filter pressing and decohesion, observed during vertebral body injection that resulted in bone instability due to low mechanical strength as well as long setting times of the cements [673]. This happens due to not only poor mechanical properties of the self-setting formulations but also some difficulties of filling vertebral bodies. In order to maintain a good cohesion and reduce filter pressing, the calcium orthophosphate formulations need to be more viscous (hence, less injectable) [4,5]. For example, they might be modified by addition of polysaccharides [120,132,380,381,382,383] and/or gelatin [317,384,385,386,387,388,389].

Another type of concerns has been raised that the use of self-setting calcium orthophosphate formulations for the augmentation of fractured and osteoporotic bones might aggravate cardiovascular deterioration in the event of pulmonary cement embolism by stimulating coagulation [674]. To investigate these potential problems, 2.0 mL of either calcium orthophosphate or polymethylmethacrylate (PMMA) cement were injected intravenously in 14 sheep. Intravenous injection of calcium orthophosphate cement resulted in a more severe increase in pulmonary arterial pressure and decrease in arterial blood pressure compared to the PMMA cement. Disintegration of the calcium orthophosphate cement seemed to be the reason for more severe reaction that represents a risk of cardiovascular complications. The authors concluded that further research efforts should aim at improving cohesion of self-setting calcium orthophosphate formulations in an aqueous environment for future clinical applications such as vertebral body augmentation [674].

The third type of concerns is related to inflammation and other adverse reactions from the surrounding tissues. Although such cases are rare, all of them must be considered in differential diagnosis of the side effects [581,582,675,676,677,678]. For example, there was a patient, who experienced an allergic reaction to Biopex^®^ [677]. A patch test was performed and a positive reaction to magnesium orthophosphate was obtained. Since Biopex^®^ contains magnesium orthophosphate, that case was diagnosed as an allergic reaction. Two publications [581,582] have been described above in Section 8.2. In addition, there are cases, such as cochlear implantation surgery [678], in which self-setting calcium orthophosphate formulations appear to be unsuitable.

To conclude the biomedical part of this review, one should mention that, although the long-term outcomes are still poorly documented, currently there are no doubts concerning a very great potential of the clinical applications of self-setting calcium orthophosphate formulations for healing of bone and dental defects. For example, a bioresorbable calcium orthophosphate cement was once found to be a better choice, at least in terms of the prevention of subsidence, than autogenous iliac bone graft for the treatment of subarticular defects associated with unstable tibial plateau fractures [679]. Furthermore, BoneSource^TM^ was found to be safe and effective when used to fill traumatic metaphyseal bone voids and appeared to be at least as good as autograft for treatment of these defects [680]. However, in other studies, autologous cancellous grafts were demonstrated to lead to a significantly better bone regeneration compared to the application of calcium orthophosphate granules produced from a self-setting calcium orthophosphate formulation after 6 weeks [681]. As this text is intended to be read mainly by chemists and materials researchers, the biological, medical and clinical aspects of self-setting calcium orthophosphate formulations have not been discussed in many details. For additional biomedical details, the interested readers are referred to other papers and reviews [22,46,52,53,160,539,544,667].

## 9. Non-Biomedical Applications

Since a non-biomedical topic is beyond the general subject of current review, this section is brief. In literature, there are some reports on brushite cement-based biosensors, one for phenol detection by combing the cement with the enzyme tyrosinase [682] and another for the detection of glucose using the enzyme glucose oxidase [683]. Both biosensors have faster signaling and higher sensitivity than traditional biosensor systems based on polymeric or clay matrices, opening up many possibilities for the future development of these devices.

## 10. Recent Achievements and Future Developments

As the self-setting calcium orthophosphate formulations represent an intriguing group of new biomaterials for bone augmentation and reconstruction, there is a great potential for further improvement of their properties, in which the ideal characteristics (Table 5) should be approached by manipulations with the chemical composition, powder particle size and distribution, as well as by means of various additives. Several commercial formulations have been already approved for the clinical applications (Table 2 and Table 3). New compositions of both apatite- and brushite-forming self-setting formulations are expected to appear in the market soon. The forthcoming commercial products will need to be improved in order to take the advantage of a variety of possibilities offered by the self-setting properties. New formulations will include: (i) injectable and open macroporous compositions to optimize their osteoconduction [317]; (ii) formulations containing only one calcium orthophosphate (single-phase powders) [220] and (iii) drug-loaded and hormone-loaded formulations for the treatment of bone diseases [46,52,53]. Furthermore, incorporation of autologous or allogenic osteo-progenitor cells into the self-setting formulations will be favorable [684,685,686]. Obviously, the first two directions deal with both chemistry and material science, while the last two directions are more related to biology and medicine.

Concerning the material point of view, an innovative approach of so-called ready-to-use self-setting formulations was introduced relatively recently. The concept was shown to work with both single-phase calcium orthophosphate powders and mixtures of several components on both brushite- and apatite-forming formulations. For example, the ready-to-use formulations can be obtained by stabilizing the calcium orthophosphate reactants as separated liquid or pasty components, with at least one of them containing an aqueous liquid, which is needed to initiate the setting reactions after mixing. Usually, such formulations consist of two injectable pastes to be mixed together and injected at the time of implantation [687,688]. The preparation process is fast and reproducible since two liquid phases can be mixed more homogeneously than powder with liquid as performed for conventional self-setting formulations. This strategy allows usage of dual chamber syringes equipped with a mixing device (e.g., by a static twin-chambered mixer incorporated in the injection cannula that allows injection of the paste immediately after mixing), meaning reduced paste processing/handling time, lesser contamination risks, enhanced reproducibility and immediate injection of the mixture into the bone defects [689]. In such formulations, a wide range of possibilities appears by changing the calcium orthophosphate components. Furthermore, such formulations can also be modulated by adjoining different additives as setting retardants, polymeric adjuvants, visco-enhancing agents, suspension stabilizers, osteoinductive agents, radio-opaque fillers or macropore-forming agents [371,434]. Nevertheless, this approach is limited to acid-base formulations only [230].

**Table 5:** Major advantages and disadvantages of the self-setting calcium orthophosphate formulations [52,53,228].

Advantages	Disadvantages
Self-setting ability *in vivo*. Good injectability that allows cement implantation by minimally invasive surgical techniques, which are less damageable than the traditional surgical techniques. Good osteoconductivity and occasional osteoinductivity: the initial biological properties of the hardened cements are similar to those of CDHA or brushite. Can be replaced by newly formed bone after a period of time (osteotransductivity). Moldability: the perfect fit to the implant site, which assures good bone-material contact, even in geometrically complex defects. Excellent biocompatibility and bioactivity. No toxicity. Low cost. Ease of preparation and handling. Setting at body temperature. Form chemical bonds to the host bone. Clinically safe materials in their powder components. Can be used to deliver antibiotics, anti-inflammatory drugs, growth factors, morphogenic proteins, *etc*. at local sites, which are able to stimulate certain biological responses. *	Mechanical weakness: limited use due to potential collapse of material followed by soft tissue formation instead of bone formation (loaded areas). Until cements with adequate shear strength are available, most complex fractures that can be repaired with cement also will require metal supports. Can be washed out from surgical defect if excess of blood. Lack of macroporosity (especially interconnected pores), which prevents fast bone ingrowth and the cements degrade layer-by-layer from the outside to the inside only. The *in vivo* biodegradation of many formulations is slower than the growth rate of a newly forming bone.

* Further studies are necessary.

Another preparation approach of the ready-to-use self-setting formulations comprises a water-reactive paste such as a mixture of TTCP and DCPD powders dispersed in a nonaqueous but water-miscible liquid (e.g., glycerol, polyethylene glycol, *N*-methyl-2-pyrrolidone) + a gelling agent (e.g., hydroxypropylmethylcellulose, carboxymethylcellulose, chitosan, sodium alginate) + a hardening accelerator (e.g., MCPM, Na_2_HPO_4_, tartaric, malic, malonic, citric or glycolic acids) to form a stable paste that can be directly injected into bone defects [690,691,692,693,694,695,696,697]. In literature, this type of self-setting pastes is called “premixed calcium phosphate cements” (occasionally referred to as PCPC), in which the paste preparation is done under defined conditions, while the pastes remain stable during storage and harden only after placement into the defect. The pastes can be obtained of different consistencies, from low viscosity ones to putty-like plastic pastes [146,147,148]. Setting occurs *in vivo* upon a contact with body fluids or *in vitro* in a physiological solution. This approach eliminates the powder-liquid mixing stage during surgery, which might improve the formulation performance. Besides, it allows shortening the surgical time and the risk of operator-induced error is considerably reduced. Unfortunately, the setting reaction of the premixed formulations is difficult to control and the mechanical properties of the hardened calcium orthophosphates are poor. Besides, such formulations must be protected from the environmental moisture during storage [698,699]. Furthermore, little attention has been paid to the problem that the presence of water impurities in the non-aqueous liquid and/or the powdered solid can compromise the stability of the paste. The composition of some premixed calcium orthophosphate formulations might be found in literature [371].

The earliest premixed self-setting calcium orthophosphate formulations were formed apatite as the final product, had a setting time of longer than 1 h and a low mechanical strength [690]. Afterwards, improved formulations have been developed. They exhibited a rapid setting when immersed in a physiological solution, yielding a hardened bioceramics with a higher mechanical strength, approached the reported strengths of sintered porous HA implants and cancellous bone [691,692,693]. Brushite-forming premixed self-setting formulations have been introduced as well [687,688,698,700,701,702]; they have shorter setting times then the aforementioned apatite-forming ones. In addition, studies appeared on preparation of the premixed monetite-forming formulations [269,270], as well as on premixed macroporous calcium orthophosphate scaffolds reinforced by slow-dissolving fibers (in other words, premixed macroporous concretes) [369]. Furthermore, antimicrobial properties might be provided as well [703,704].

The third approach to manufacture the ready-to-use self-setting calcium orthophosphate formulations applies very low temperatures [36]. According to this approach, powder and liquid components of the self-setting formulations are mixed and the prepared pastes are immediately frozen. Thus, premixed frozen calcium orthophosphate “slabs” are obtained, which are stored in freezers or even in liquid nitrogen. By freezing, the setting reactions are slowed down or even inhibited (this depends on the temperature) but when the formulations have to be applied, the “slabs” are defrosted and the softened pastes are molded by hands at ambient temperatures. When frozen and stored at *t* = −80 °C or less, significant degradation in compression strength did not occur for the duration of the study (28 days). Interestingly, that in the case of the brushite-forming formulations prepared from a combination of β-TCP with 2 M H_3_PO_4_ solution, freezing the paste had the effect of increasing mean compressive strength fivefold (from 4 to 20 MPa), which was accompanied by a reduction in the setting rate of the cement. This strength improvement was attributed to a modification of crystal morphology and a reduction in damage caused to the cement matrix during manipulation [36].

A lack of macropores is a substantial disadvantage of many current self-setting calcium orthophosphate formulations [311]. As a result, biodegradation takes place layer-by-layer on the surface, from outside to inside. To solve this problem, various types of porogens are used [337,338,339,340,341,342,343,344,345,346,347,348,349,350,351,352,353,354,355,356,357,358,359,360,361]. Using a hydrophobic liquid instead of soluble particles could be an alternative. At the turn of the millennium, an open macroporous structure was obtained using a mixture of oil and a self-setting paste [705]; however, since than no research papers on this subject have been published. Besides, by means of surfactants, air bubbles might be incorporated into the bulk of the formulations [330]. Unfortunately, the mechanical strength and porosity are conflicting requirements. As porosity of the calcium orthophosphate formulations appears to be of paramount importance to achieve an excellent bioresorbability, other experimental approaches have to be developed [706].

In the case of calcium orthophosphate reinforced formulations and concretes, future studies could combine in one formulation porogens and biodegradable fibers of different shapes and dissolution rates to form after *in vivo* hardening scaffolds with sustained strength. In such a system, one porogen is quickly dissolved, which creates macropores to start a bone ingrowth process, while the second type of fibers provides the required strength to the implant. After significant bone ingrowth into the initial pores increased the implant strength, the second set of fibers would then be dissolved to create additional macropores for bone ingrowth [362]. Such complicated formulations have already been developed. For example, chitosan, sodium orthophosphate and hydroxypropylmethylcellulose were used to render calcium orthophosphate formulations fast setting and resistant to washout, while absorbable fibers and mannitol porogen were incorporated for strength and macropores, respectively. Both strength and fracture resistance of this concrete were substantially increased and approached those values for sintered porous HA implants [707]. Turning on a bit of imagination, one might predict development of polymeric forms of drugs (already available [708,709]), hormones, growth factors, *etc*. (e.g., prepared by either incorporation into or cross-linking with either water-soluble or bioresorbable polymers). Coupled with reinforcing biodegradable fibers and porogens, such types of “healing fibers” might be added to self-setting calcium orthophosphate formulations, which not only will accelerate the remedial process, but also will allow simultaneous improvement in both their strength and injectability. In addition, graded structures are possible. For example, a layered structure was designed by combining a macroporous layer of calcium orthophosphate cement with a strong fiber-reinforced calcium orthophosphate concrete layer. The rationale for such construction was for the macroporous layer to accept tissue ingrowth, while the fiber-reinforced strong layer would provide the needed early-strength [710].

Stability (insolubility) in normal physiological fluid environment and resorbability under acidic conditions produced by osteoclasts appears to be among the most important *in vivo* characteristics of modern types of calcium orthophosphate bioceramics. For some clinical applications, such as cranioplasty, a relatively slow resorption and replacement by bone is quite acceptable, whereas in other applications, such as periodontal bone defects repair, sinus lift, *etc*., the ability of the hardened formulations to be replaced quickly by bone is crucial. Experimental results suggest that a number of parameters of the self-setting calcium orthophosphate formulations, such as the Ca/P ionic ratio, carbonate content, ionic substitution, crystallinity, *etc*. might affect the dissolution characteristics in slightly acidic solutions. This gives an opportunity to formulate compositions, possessing different resorption rates, which is suited for different biomedical applications [166,167].

Furthermore, the discovery of self-setting calcium orthophosphate formulations has already opened up new perspectives in synthesis of bioceramic scaffolds, possessing sufficient mechanical properties and suitable for tissue engineering purposes [339,343,344,462]. In the past, strong scaffolds could only be manufactured by the sintering route at elevated temperatures [711]. Therefore, until recently it was impossible to produce resorbable preset low-temperature hydrated 3D bioceramics for various applications, e.g., scaffolds and granules, from low-temperature calcium orthophosphates, such as ACP, DCPA, DCPD, OCP and CDHA. Currently, using the appropriate techniques (e.g., 3D powder printing [658,712,713,714], open macroporous 3D scaffolds [189,338,371,377,378,379,462,714,715,716,717] and/or other objects [718,719] consisting of the aforementioned low-temperature phases (currently, excluding ACP and OCP) can be produced via a cementitious reaction; thus, dramatically widening the biomedical applications of low-temperature calcium orthophosphates. This type of bioceramics could be very promising for tissue engineering applications and, among them, CDHA is of a special interest due to its chemical similarity to bone material and a large specific surface area.

Nevertheless one should stress, that the most promising direction of the future developments of the self-setting calcium orthophosphate formulations is obviously seen in their functionalization by incorporation of (or impregnation by) various hormones, growth factors, drugs, other bioorganic compounds, as well as incorporation of living cells and/or other tiny biological objects [720,721,722,723,724,725,726,727,728,729,730,731,732]. For example, silk fibroin can regular the mineralization process and bond with HA to form fibroin/HA nanodimensional biocomposites with increased gelation properties and, thus, it can be used as an additive to improve cohesion of calcium orthophosphate formulations and decrease a risk of cardiovascular complications in its application in veterbroplasty and kyphoplasty [727].

While the simplicity in the processing of self-setting formulations encourages the incorporation of cells, the principal difficulty remains to ensure cell survival. The harsh environment in terms of pH and high ionic strength together with the high stiffness achieved upon hardening can be thought as the principal threats for cell endurance. The initial attempts have already been performed but without a great success yet. For example, researchers have already found that unset calcium orthophosphate formulations might have toxic effects when placed on cell monolayers, while the set formulations are biocompatible for the same type of cells (MC3T3-E1 osteoblast-like cells were tested). A gel encapsulation in alginate beads was found to be a possible solution to protect living cells for seeding into self-setting pastes [685,733]. *In vitro* cytotoxic effect of α-TCP-based self-setting formulation was also observed [734]. In light of these results, the encapsulation approach [352] could potentially be used to seed a patient’s *ex vivo* expanded stem cells into the formulations to create osteoinductive bone grafts those could be used to treat that patient. However, this becomes more related to tissue engineering and biology, rather than to chemistry and material science. A first possibility would be designing self-setting formulations that have setting reactions close to the physiological pH or by adding additives into the self-setting pastes able to neutralize the acidic or basic ions released during the chemical reactions.

In addition, besides the aforementioned chemical, material and biomedical improvements of the self-setting calcium orthophosphate formulations, one should not forget on a better design of both the mixing equipment and delivery (injection) techniques. As an example, the interested readers are referred to a new cannula to ease cement injection during vertebroplasty [735]; however, this subject is beyond the scope of current review.

Finally, one should not forget on the recent progress in self-setting formulations used as construction materials. Due to the ceramic nature, industrial concretes are very sensitive to crack formation because of their limited tensile strength. Therefore, self-healing formulations are developed [736,737,738,739]; presumably, some of these principles and approaches might be applied for the biomedical formulations as well.

## 11. Conclusions

Thus, among the diverse range of bone replacing biomaterials, self-setting calcium orthophosphate formulations undoubtedly represent a distinct group because they are relatively simple biomaterials formed by combining of a calcium orthophosphate mixture with an aqueous solution. However, they symbolize an important breakthrough in the field of bone repair bioceramics, since they offer the possibility of obtaining thermally unstable calcium orthophosphates in a monolithic form at room or body temperature by means of a cementation reaction. This particular fabrication technique implies that the self-setting formulations are moldable and therefore can adapt easily to the bone cavity providing a good fixation and the optimum tissue-biomaterial contact, necessary for stimulating bone ingrowth into them and their subsequent osteotransduction [46].

Unfortunately, the perfect grafting material does not exist. The self-setting calcium orthophosphate formulations are not an exception to this statement. While possessing excellent biological properties (osteoconduction and, occasionally, osteoinduction), adequate setting time, excellent moldability and the capability to deliver different bone-enhancing proteins/antibiotics at a local level, unfortunately, the biomaterial lacks adequate mechanical properties for applications other than non-loaded surgical sites (see Table 5 for other details). Nevertheless, even in its present state, the self-setting calcium orthophosphate formulations appear to be suitable for a number of applications. They can be injected into osteoporotic bone to reinforce it or can be used to make granules and blocks out of low-temperature calcium orthophosphates. Several types of the self-setting formulations are now on the market (Table 2 and Table 3), while scaffolds made of low-temperature calcium orthophosphates are being tested. The use of slightly different chemical compositions and various dopants affects both the setting time and tensile strength that enables further improvements. In addition, new trials are conducted with the reinforced formulations and concretes, which represent additional attempts to improve the existing products.

It is anticipated that the use of self-setting calcium orthophosphate formulations will enable a faster and more aggressive rehabilitation, as the strength of the hardened concretes makes it possible to allow full weight-bearing earlier than when bone graft is used. Although, preliminary clinical trials have already confirmed the great potential of this novel therapeutic product, the self-setting calcium orthophosphate formulations need to be improved further; in particular, their bioresorption needs to be accelerated as well as their injectability and mechanical properties need to get better. Besides, extra clinical studies are required to define the most appropriate indications and limitations of calcium orthophosphate formulations for fracture repair.

In the author’s humble opinion, mentioning the James M. Anderson’s opinion on the history of biomaterials field would be the best way to conclude this subject. According to Anderson, within 1950–1975 researchers studied biomaterials, within 1975–2000 they studied biomaterials and since 2000 the time for BIOmaterials has been coming [740]. Here, the capital letters emphasis the major direction of the research efforts in the complex subject of biomaterials. As the real history of self-setting calcium orthophosphate formulations started only in 1983, the aforementioned periods were shifted along the time scale. Certainly, the biomaterials-epoch for the self-setting formulations is over (every possible combination of calcium orthophosphates has been already studied and tested), while the biomaterials-era (where cells are the key factor) is just at the very beginning. Most likely, current state-of-the-art of the self-setting calcium orthophosphate formulations corresponds to the final stages of the biomaterials-phase with an approximately equal contribution of the biological and materials directions. Therefore, still there is much room for versatile ideas and approaches.
